# Rapid response of lichen planus to baricitinib associated with suppression of cytotoxic CXCL13^+^CD8^+^ T cells

**DOI:** 10.1172/JCI179436

**Published:** 2024-11-14

**Authors:** Angelina S. Hwang, Jacob A. Kechter, Tran H. Do, Alysia N. Hughes, Nan Zhang, Xing Li, Rachael Bogle, Caitlin M. Brumfiel, Meera H. Patel, Blake Boudreaux, Puneet Bhullar, Shams Nassir, Miranda L. Yousif, Alyssa L. Stockard, Zachary Leibovit-Reiben, Ewoma Ogbaudu, David J. DiCaudo, Jennifer Fox, Mehrnaz Gharaee-Kermani, Xianying Xing, Samantha Zunich, Emily Branch, J. Michelle Kahlenberg, Allison C. Billi, Olesya Plazyo, Lam C. Tsoi, Mark R. Pittelkow, Johann E. Gudjonsson, Aaron R. Mangold

**Affiliations:** 1 Mayo Clinic Arizona Department of Dermatology, Scottsdale, Arizona, USA.; 2 University of Michigan Department of Dermatology, Ann Arbor, Michigan, USA.; 3 Mayo Clinic Department of Quantitative Health Sciences, Scottsdale, Arizona, USA.; 4 Mayo Clinic Department of Laboratory Medicine and Pathology, Scottsdale, Arizona, USA.; 5 University of Michigan Department of Internal Medicine, Rheumatology, Ann Arbor, Michigan, USA.

**Keywords:** Dermatology, Drug therapy, T cells, Th1 response

## Abstract

**BACKGROUND:**

Cutaneous lichen planus (LP) is a recalcitrant, difficult-to-treat, inflammatory skin disease characterized by pruritic, flat-topped, violaceous papules on the skin. Baricitinib is an oral Janus kinase (JAK) 1/2 inhibitor that interrupts the signaling pathway of IFN-γ, a cytokine implicated in the pathogenesis of LP.

**METHODS:**

In this phase II trial, 12 patients with cutaneous LP received 2 mg daily baricitinib for 16 weeks, accompanied by in-depth spatial, single-cell, and bulk transcriptomic profiling of pre- and posttreatment samples.

**RESULTS:**

An early and sustained clinical response was seen, with 83.3% of patients responsive at week 16. Our molecular data identified a unique, oligoclonal IFN-γ, CD8^+^, and CXCL13^+^ cytotoxic T cell population in LP skin and demonstrated a rapid decrease in IFN signature within 2 weeks of treatment, most prominently in the basal layer of the epidermis.

**CONCLUSION:**

This study demonstrates the efficacy and molecular mechanisms of JAK inhibition in LP.

**TRIAL REGISTRATION:**

NCT05188521

**FUNDING:**

Eli Lilly, Appignani Benefactor Funds, 5P30AR075043, Mayo Clinic Clinical Trials Stimulus Funds.

## Abstract 2

This study demonstrates the safety and efficacy of baricitinib and identifies a novel T-cell subset as a major source of IFN-γ in cutaneous lichen planus.

## Introduction

Lichen planus (LP) is a chronic inflammatory condition typified by purple, polygonal, pruritic papules, and plaques (1). LP can affect any tissue derived from the ectoderm, including the skin, nails, and mucous membranes. Cutaneous LP affects 1%–2% of the general population and has a substantial impact on quality of life (QoL) primarily due to intense pruritus or pain (2). Certain subtypes, such as hypertrophic and mucosal LP, are symptomatic, chronic, and refractory to treatment (3, 4).

Treatment of LP is challenging, and therapeutic options have remained largely stagnant. First-line therapy is commonly topical steroids. Other therapies include topical calcineurin inhibitors, oral retinoids, methotrexate, and oral or intralesional steroids (1). However, optimal results are rarely achieved, and long-term use of these medications can lead to considerable adverse effects. To date, no disease-specific medications have been developed despite the need for therapeutics with a more favorable side-effect profile and for recalcitrant cases.

LP is a T cell–mediated disease with IFN-γ established as a key mediator in pathogenesis (5). This cytokine attracts lymphocytes and plasmacytoid dendritic cells to the epidermis and stimulates the interaction between keratinocytes (KCs) and lymphocytes (6, 7). CD4^+^ T cells release IFN-γ, leading to CD8^+^ T cell stimulation and Th1 inflammatory response propagation (8, 9). KCs primed by IFN-γ have increased susceptibility to the cytotoxic effects of activated CD8^+^ T cells (8).

IFN-γ signals through the Janus kinase (JAK) signal transducer and activator of transcription (STAT) pathway, and recent studies of LP have highlighted remarkable responses to JAK inhibitors (10–15). An exploratory, open-label study of topical ruxolitinib (JAK-1/2 inhibitor) resulted in significant reductions in total lesion count and modified composite assessment index lesion severity (mCAILS) scores, achieving therapeutic response in 83% of treated lesions (16). Baricitinib is an oral JAK-1/2 inhibitor that prevents the phosphorylation of STATs and the subsequent signaling of IFN-γ. Case reports and retrospective studies have reported successful baricitinib treatment for nail LP, oral LP, and lichen planopilaris (12, 17, 18). In this first-in-human trial, we conducted an open-label, single-arm study of baricitinib in cutaneous LP and defined the molecular profile and signature of disease using bulk, spatial, and single-cell RNA-sequencing (scRNA-Seq) on pre- and posttreatment specimens.

## Results

### Patients.

**Figure 1:**
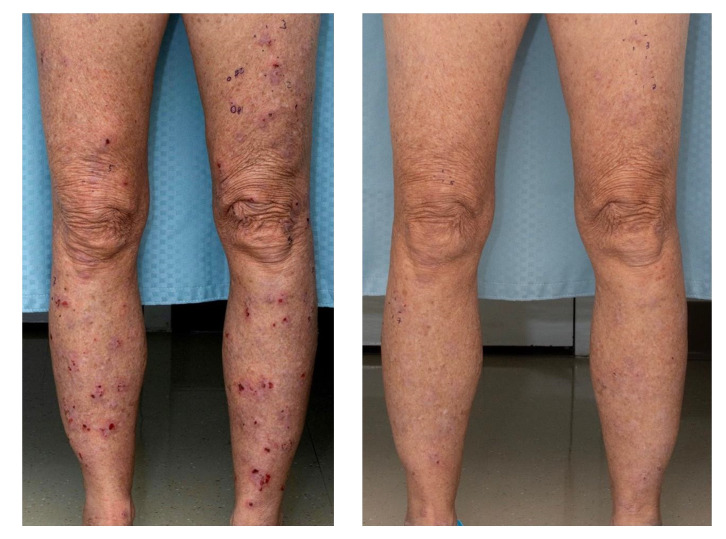
Example image of cutaneous LP response to baricitinib (week 0 versus week 16).

A total of 12 patients with a mean age of 63.6 (SD 13.6) years were enrolled. The majority (*n* = 11, 91.7%) were female and identified as White (*n* = 9, 75.0%). There was one male patient (8.3%), and 25% of the study population identified as Black or African American (*n* = 1, 8.3%) or Hispanic or Latino (*n* = 2, 16.7%). The mean disease duration across all patients was 26.5 months (SD 30.8). All patients had LP refractory to prior therapy, with 91.7% failing topical steroids, 41.7% failing oral and intramuscular steroids, 8.3% failing methotrexate, and 8.3% failing topical calcineurin inhibitors. Hypertrophic LP was seen in 5 (41.7%) patients (Figure 1 and Supplemental Figure 1; supplemental material available online with this article; https://doi.org/10.1172/JCI179436DS1), and 2 (16.7%) had mucosal involvement (Table 1 and Supplemental Figure 1); however, classic LP was the predominant form in all patients. Demographics and outcomes on an individual patient level are summarized in Table 1.

The average affected body surface area (BSA) was 4.9% at baseline (SD 3.7), with a mean of 151.9 total body LP lesions per patient (range 4–600) (Table 2). The mean baseline mCAILS score was 12.3 (SD 3.2), and the overall Skindex-16 (19, 20) was 59.0 (SD 22.1). Pruritus numeric rating scale (NRS) and pruritus visual analog scale (VAS) scores were 7.2 (SD 2.4) and 6.6 (SD 1.6), respectively, with 91.7% of patients rating their level of itch as moderate/severe on the pruritus verbal rating scale (VRS). The baseline pain NRS score was 7.7 (SD 1.7).

### Efficacy.

At week 16, 10 of 12 (83.3%; 95% CI: 51.6%–97.9%) patients demonstrated treatment response, achieving physician global assessment (PGA) scores of 0 to 3, with 50% or greater score reduction (Figure 1 and Table 2). Five of the 10 treatment-responsive patients had a PGA of 0 (completely clear), and 5 had a PGA of 1 (almost clear) (Supplemental Table 1 and Supplemental Figure 2). Improvement in PGA was observed as early as week 1 in 37.5% of patients and in 100% of patients by week 12. Treatment effects were sustained at week 20 (4 weeks off therapy), with all patients demonstrating continued response.

Improvements were seen across all secondary measures at week 16 (Table 2). The mean total body lesion count decreased to 17.1 (SD 33.5; *P* = 0.002), and the mean affected BSA decreased to 1.0 (SD 2.5; *P* = 0.002). Compared with a baseline score of 7.2 (SD 2.4), pruritus NRS decreased to 1.8 (SD 3.2; *P* = 0.003) (Supplemental Figure 3), pruritus VAS decreased from 6.6 (SD 1.6) to 1.7 (SD 3.0; *P* = 0.003), and pain NRS decreased from 7.7 (SD 1.7) to 1.9 (SD 3.2; *P* = 0.005) (Supplemental Figure 4). Pruritus NRS improvement from baseline (NRS4) and pain NRS4 was achieved in 75.0% and 66.7% of patients, respectively. The overall Skindex-16 score decreased from baseline to week 16 by a mean of 37.3 (SD 18.3; *P* = 0.008), accompanied by decreases in each Skindex subscore: symptom, –12.4 (SD 5.8; *P* = 0.005); emotional, –20.9 (SD 9.4; *P* = 0.003); and functional, –5.5 (SD 5.6; *P* = 0.012). Results from the per-protocol analysis, with the population defined as patients who completed 16 weeks of baricitinib, were consistent with the results of the intention-to-treat (ITT) analysis (Supplemental Table 2).

### Dose escalation.

Five of 6 eligible patients participated in the dose-escalation period. All 5 patients completed an additional 12 weeks of treatment with 4 mg baricitinib daily. At the primary endpoint of week 16, corresponding with the start of dose escalation, 80.0% of patients had PGA grade 1, and 20% had PGA grade 4. After 12 weeks of 4 mg baricitinib daily, 60.0% of patients were completely clear of disease (PGA grade 0), 20.0% were almost clear (PGA grade 1), and 20.0% had slight improvement (PGA grade 4) (Table 3). Only one patient remained treatment responsive upon reevaluation after 4 weeks off therapy (Supplemental Table 3).

### Safety.

There was a total of 12 adverse events (AEs), with only one mild AE that was deemed probably related to the study drug (absolute neutrophil count 0.78 × 10(9)/L) (Supplemental Table 4). Most AEs were mild or moderate (58.3% and 25.0%, respectively). No AEs led to the discontinuation of baricitinib.

### Molecular profiling of lesional and nonlesional tissue.

**Figure 2:**
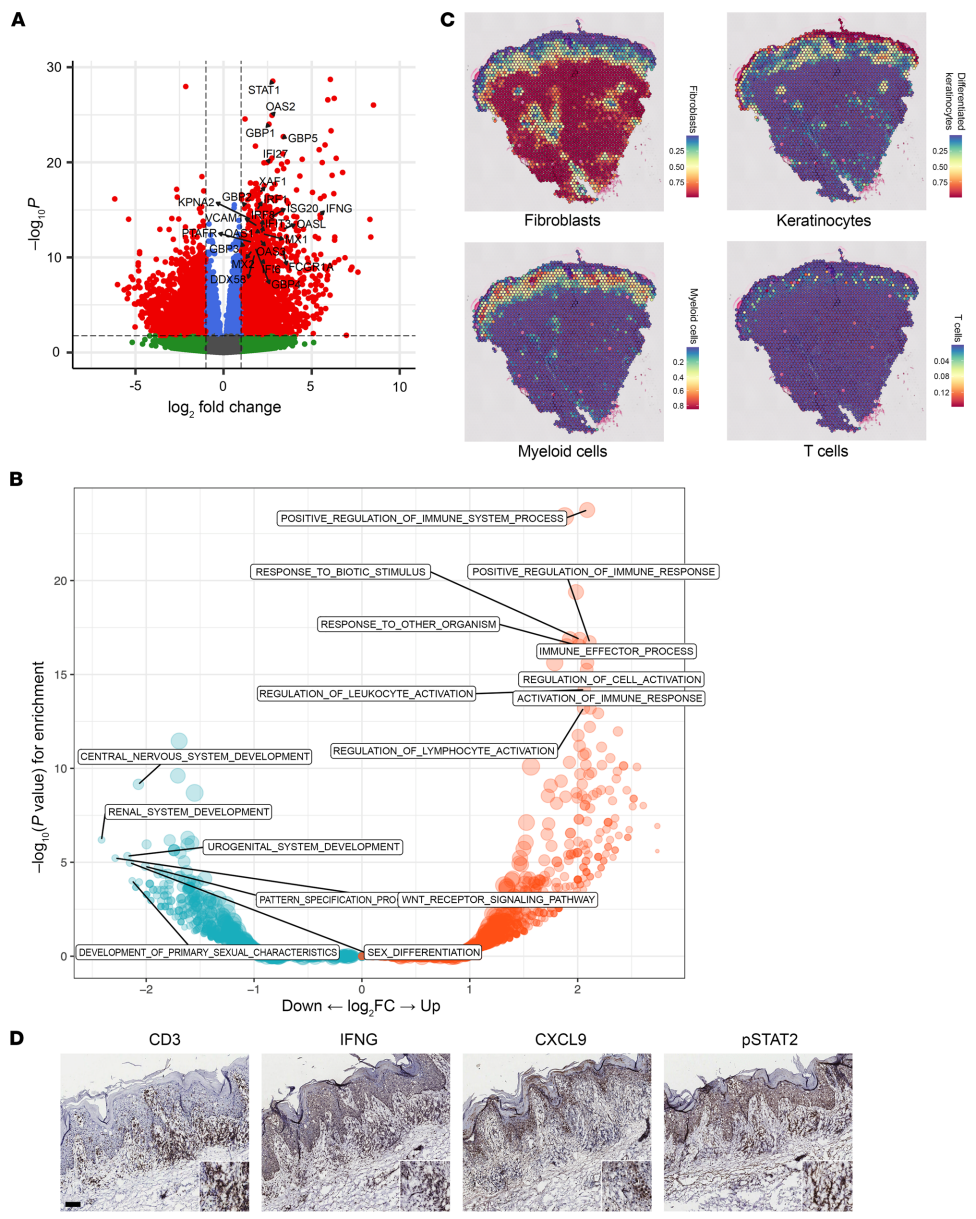
LP is an IFN-driven disease process. (**A**) Volcano plot of bulk RNA-Seq data comparing lesional versus nonlesional LP skin at day 0 (*n* = 10 and 9, respectively) (red color shows FDR < 0.05 and log_2_ FC > 1 or less than negative 1, blue is FC < 1 and less than negative 1 and FDR < 0.05), green is FC > 1 or less than negative 1)and FDR > 0.05). (**B**) Enriched GO categories in DEGs between lesional versus nonlesional LP skin (red and blue colors represent enriched GO categories among increased versus decreased DEGs, respectively, *P* < 0.05). (**C**) Cellular deconvolution of fibroblasts, KCs, myeloid cells, and T cells on the Visium 10X spatial expression platform (representative of *n* = 9). (**D**) IHC of the T cell marker CD3, pSTAT2, IFN-γ, and CXCL9 (representative of *n* = 9). Scale bar: 100 μm.

Whole transcriptomic analysis using bulk RNA-Seq was performed on lesional and nonlesional skin prior to therapy (*n* = 11, 12, respectively). Differential expression (DE) analyses were conducted to identify differentially expressed genes (DEGs) (FDR ≤ 0.05, and log_2_[fold change (FC)] ≥ 1). The DE analysis for lesional versus nonlesional LP skin at day 0 revealed 3,524 DEGs, with 1,683 increased and 1,841 decreased compared with nonlesional LP skin. The most prominent DEGs in lesional LP skin were IFN-stimulated genes (ISGs), including *STAT1* (FC = 6.9, FDR = 8 × 10^–25^), *OAS2* (FC = 6.7, FDR = 9 × 10^–22^), *MX1* (FC = 4.8, FDR=1 × 10^–10^), and *ISG20* (FC = 6.6, FDR = 2.9 × 10^–12^), with *IFNG* (FC = 44.6, FDR = 2.0 × 10^–12^) being the most prominent interferon member expressed (Figure 2A and Supplemental Table 5). Enriched Gene Ontology (GO) categories at day 0 showed enrichment for immune-effector process, response to virus, interferon signaling, and antigen processing and presentation (Figure 2B).

To assess cellular architecture in LP, we performed spatial RNA sequencing (spRNA-Seq) using the 10X Visium platform on lesional LP biopsies from 9 patients both at baseline and at 2 weeks. After quality control (see Methods), we identified, on average, 1,530 spots with an average of 37,398 reads per spot, corresponding to 934 genes. The spatial data showed transcriptomic changes consistent with dense infiltration of myeloid and T cells in the upper dermis, right below and adjacent to the epidermis (Figure 2C). The increased T cell infiltration, IFN-γ expression, and enriched IFN responses were validated by immunohistochemistry of lesional LP skin (Figure 2D).

**Figure 3:**
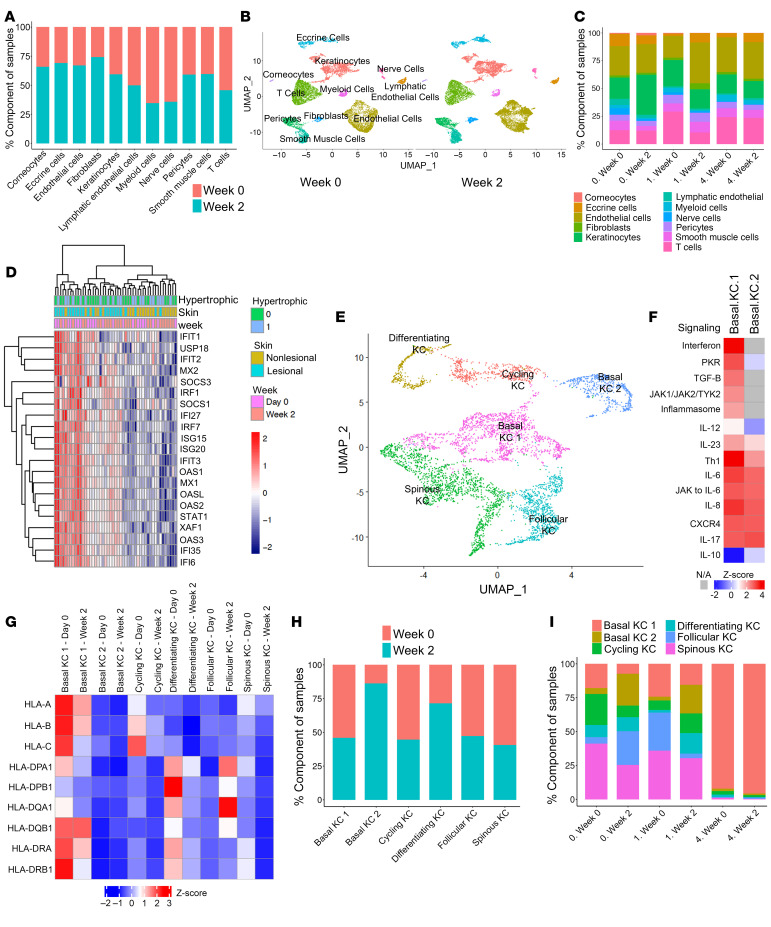
Cellular composition of LP and effect of baricitinib treatment. (**A**) Cell proportions at baseline and week 2. (**B**) Single-cell data from baseline (day 0) and at week 2 in the patient cohort (*n* = 9, 9). (**C**) Proportion of cell type with PGA score based on week 16 response in which 0 means total clear (*n* = 3), 1 means almost clear (*n* = 5), and 4 means no improvement (*n* = 1) at baseline (week 0) and week 2 of treatment. (**D**) Changes in gene expression in interferon signature genes at baseline and week 2. (**E**) Single-cell data from the LP cohort defines 6 distinct KC clusters, including 2 basal cell states. (**F**) Enriched GO categories in the 2 basal KC clusters. (**G**) Expression of MHC class I and class II molecules in the different KC compartments at different time points. (**H**) The proportion of each KC subset at baseline and week 2 of treatment. (**I**) Proportion of KC subclusters with PGA score based on week 16 response in which 0 means total clear, 1 means almost clear, and 4 means no improvement at baseline (week 0) and week 2 of treatment.

To better assess the cellular mechanisms involved in LP, we performed scRNA-Seq from baseline and week 2 lesional LP biopsies from 9 patients. After quality control (see Methods), we identified 30,825 cells with 1,668 genes and 4,781 transcripts detected per cell, from 9 donors with PGA scores of 0 (total clear, *n* = 3), 1 (almost clear, *n* = 5), and 4 (no improvement, *n* = 1) at week 16 (Supplemental Table 6). Using the unbiased clustering method from the Seurat R package (version 5.0.1), we identified 20 cell clusters and overlapped these with known canonical cell type markers to annotate 11 major cell types. We identified major cell subsets in lesional LP skin, including KCs, corneocytes, fibroblasts, endothelial cells, lymphatic endothelial cells, T cells, myeloid cells, eccrine cells, smooth muscle cells, pericytes, and nerve cells (Figure 3, A and B, and Supplemental Figure 5, A–C). All 11 major cell types were identified in all 3 groups of patient responders (PGA 0, 1, and 4), with the most notable shift showing a decrease in the proportion of KCs in patients with a PGA score of 0 and T cells in patients with a PGA score of 1 from week 0 to week 2 (Figure 3C). In contrast, there were minimal changes in the cellular composition in the single patient who did not have a clinical response (PGA score of 4) in the same time frame (Figure 3C). Accompanying these shifts in immune cell populations was a marked decrease in ISG expression within 2 weeks of treatment by bulk RNA-Seq (Figure 3D).

To understand how KCs contribute to the pathogenesis of LP and changes in their function during treatment, we subclustered KCs into basal KC, follicular KC, spinous KC, differentiating KC, and cycling KC (Supplemental Figure 6A). KCs in the basal layer of the epidermis demonstrated 2 distinct states, basal KC 1 and basal KC 2 (Figure 3E and Supplemental Figure 6, A–D), with the basal KC 1 state having enrichment for inflammatory processes, particularly interferon and JAK1/JAK2 signaling, which were absent in the basal KC 2 state (Figure 3F and Supplemental Figure 6C). The basal KC 1 state also had a marked increase in expression of MHC class I and class II molecules (Figure 3G), suggesting that these cells may be the main target of cytotoxic responses in LP. Notably, with baricitinib treatment, there was a marked shift from basal KC 1 state to basal KC 2 state (Figure 3, H and I), reflecting suppression of IFN responses (Figure 3F and Supplemental Figure 6D) and decreased antigen presentation (Figure 3G). To address changes in treatment response, data at week 16 showed a reduced proportion of basal KC 1 and an increased proportion of basal KC 2 in patients with robust treatment responses to baricitinib (PGA scores of 0 and 1) (Figure 3I), whereas in the patient with lack of response (PGA score of 4), basal KC1 remained the dominant state (Figure 3I). These results suggest that the basal KC 1 state reflects inflammatory activity in LP.

**Figure 4:**
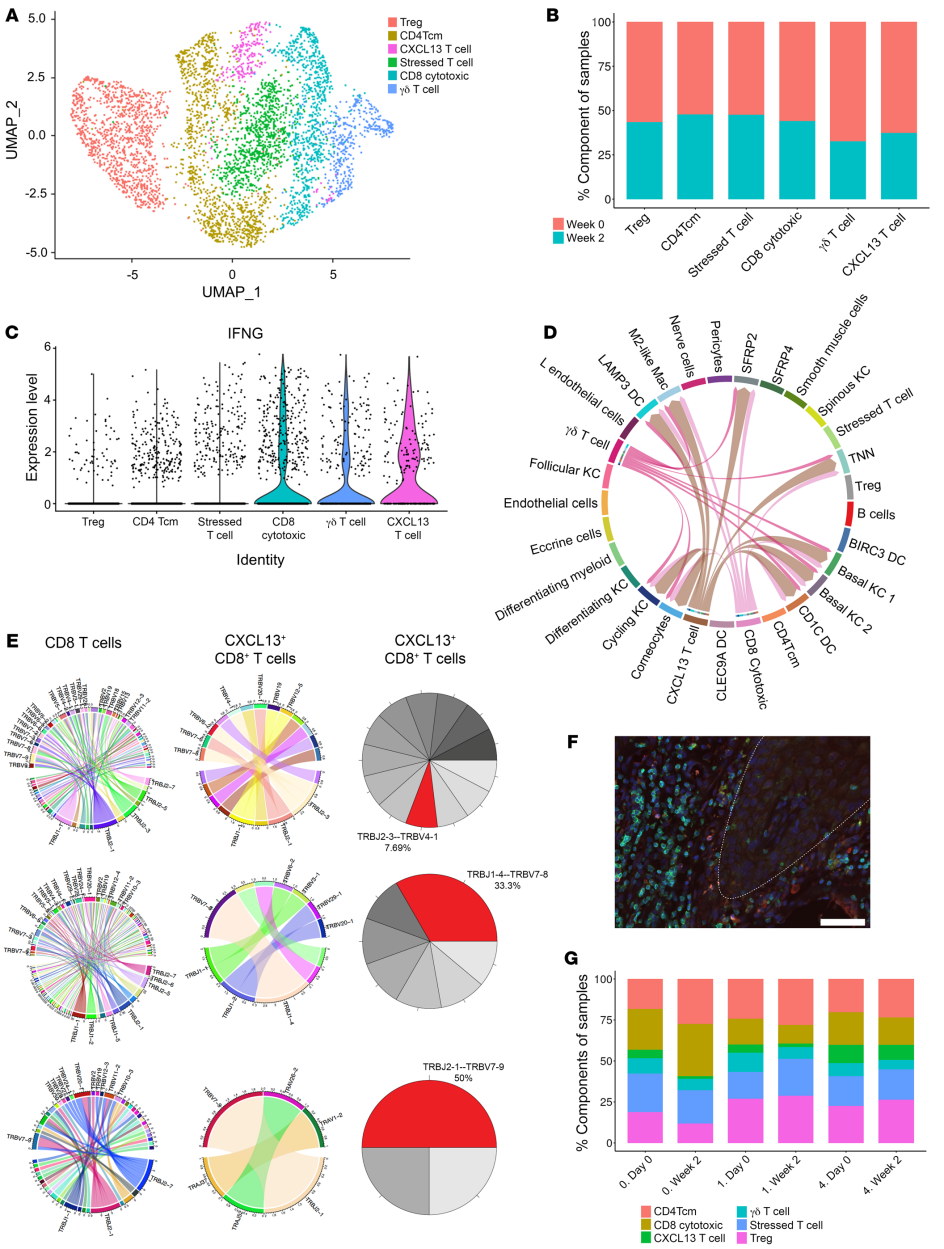
T cell function in LP. (**A**) Six T cell subsets are found in LP skin. (**B**) The proportion of T cell subsets at baseline and week 2 of treatment. (**C**) IFN-γ expression in T cell subsets in LP skin. (**D**) Type II IFN signaling network in LP skin. (**E**) Oligoclonality of CXCL13^+^CD8^+^ T cells in LP skin, showing gene expression of T cell receptor β joining and variable regions from 3 representative patients. (**F**) Immunofluorescence of CD3 (green) and CXCL13 (red) in LP skin, showing colocalization of double-positive CXCL13^+^CD8^+^ T cells adjacent to the epidermal-dermal junction (white broken line) (representative image of *n* = 3). Scale bar: 100 μm. (**G**) Proportion of T cell subclusters with PGA score based on week 16 response in which 0 means total clear, 1 means almost clear, and 4 means no improvement at baseline (week 0) and week 2 of treatment.

LP has prominent T cell infiltration, but the nature of the T cell involvement has not previously been addressed. We identified 6 subclusters of T cells in LP, including Tregs, CD4^+^ central memory T cells (CD4Tcm), “stressed” T cells (21, 22), CD8 cytotoxic T cells, γ-Δ T cells, and a CXCL13^+^ T cell population (Figure 4, A and B, and Supplemental Figure 7, A and B). CD8 cytotoxic, γ-Δ, T cell, and CXCL13^+^ T cell subsets were the major sources of *IFNG* expression in lesional LP skin (Figure 4C). Using cell-type signatures from single-cell data of LP skin, cell signatures from LP skin obtained from our scRNA-Seq dataset were integrated with spatial sequencing data from lesional LP. T cell subsets identified were localized in the upper layers of the dermis with a prominent expression of cytotoxic markers, including *GZMB*, *GZMA*, and *GNLY* (Supplemental Figure 7C). We observed a 60%–75% decrease in the proportion of CXCL13^+^CD8^+^ T cells in lesional skin from week 0 to week 2 during treatment in patients with complete or near-complete clinical response (PGA scores 0 and 1) at week 16, respectively. Meanwhile, the single patient with a minimal response (PGA score of 4) had a higher proportion of CXCL13^+^CD8^+^ T cells at baseline and only a 20% decrease of CXCL13^+^CD8^+^ T cells with baricitinib treatment (Figure 4G). Cell-cell interaction analysis revealed enriched predicted cell-cell interactions of CXCL13^+^CD8^+^ T cells with stromal cells, particularly basal KCs (Figure 4D and Supplemental Figures 8 and 9). The CXCL13^+^ T cell subset had evidence of oligoclonality in LP skin, with some clones representing up to 50% of CXCL13^+^CD8^+^ cells in some patients (Figure 4E). The CD3/CXCL13 subset was found predominantly near the basal layer of LP epidermis (Figure 4F and Supplemental Figure 10). We did not observe prominent mRNA expression of other T cell cytokines in LP, including the Th17 cytokines *IL17A*, *IL17F*, *IL22*, *IL26*, or the Th2 cytokine *IL4*. However, there was a detectable expression of *IL13* in our single-cell data, including the CXCL13^+^ subsets (Supplemental Figure 11). Notably, *IL13* mRNA expression was increased in lesional LP skin compared with nonlesional skin in our bulk RNA-Seq data (17-fold, FDR = 3.3 × 10^–8^) along with increases in the mRNA expression of the IL-4 receptor (1.7-fold, FDR = 6.4 × 10^–4^) and *IL32* (3.9-fold, FDR = 1 × 10^–8^), but no increase was in *TSLP* mRNA expression (Supplemental Table 5).

We identified 5 populations of myeloid cells (M2-like, *LAMP3*, *CD1C*, *CLEC9A*, and proliferating myeloid cells) along with a small number of B cells (Supplemental Figure 12A) and 3 major fibroblast subsets (*SFRP2*, *TNN*, and *SFRP4*) (Supplemental Figure 12B).

### Molecular profiling of peripheral blood.

We performed scRNA-Seq on 16 samples of PBMCs obtained from 10 patients in our LP cohort with PGA scores of 0 (total clear, *n* = 4), 1 (almost clear, *n* = 4), and 4 (no improvement, *n* = 1) at week 16 (Supplemental Table 7). One PBMC donor did not have a PGA score reported at week 16 due to withdrawal from the study (see Methods). Clusters were annotated manually using a curated list of marker genes. PBMC samples were differentiated into cell types, which included Tregs, pDCs, NK cells, myeloid cells, CD8^+^ T cells, CD4^+^ T cells, and B cells. Top marker genes were identified for each cell type (Supplemental Figure 13, A and B), and scRNA-Seq of PBMCs revealed no major shifts in cell populations before and after treatment (Supplemental Figure 14A). However, a decrease in ISG expression of *IFITM1*, MHC class I (*HLA-B*), class II (*HLA-DPA1*), and the cytotoxic marker *GNLY* was seen with baricitinib treatment across multiple cell populations (Supplemental Figure 14B). Biological processes that decreased with baricitinib treatment in our LP cohort included a decrease in interferon signaling (*P* = 1.5 × 10^–8^) in myeloid cells and MHC class II antigen presentation (*P* = 9.4 × 10^–4^) in CD4^+^ T cells (Supplemental Figure 14C).

## Discussion

This open-label, single-arm trial demonstrated rapid and sustained response to baricitinib in cutaneous LP. The primary outcome of PGA scores 0 to 3 (with ≥ 50% score improvement) was achieved in 83.3% of patients. Rapid improvement with baricitinib was seen, with patients showing clinical response, as assessed by PGA, as early as week 1, with 75% of patients showing a response by week 2. After 4 weeks of drug discontinuation, all patients had sustained improvement per protocol, with the majority remaining clear or almost clear of disease, thus providing key indications of the short-term persistence of the therapeutic effects of baricitinib. In patients with concomitant mucosal LP, improvements were seen in both cutaneous and mucosal LP lesions with baricitinib.

Most patients in our study had chronic, treatment-refractory LP, with over 40% having the hypertrophic variant. The mean disease duration in our study population was 26.5 months, and half had failed systemic therapy, including methotrexate and oral or intramuscular corticosteroids. All but one had previously trialed topical steroids without success. The high response rates demonstrated by this cohort highlight the therapeutic efficacy of baricitinib, even in recalcitrant LP. Similar response rates were reported with topical ruxolitinib in more limited cases of LP (16).

The dose-escalation group comprised 5 patients without complete clearance of disease at the primary endpoint, who were subsequently escalated to 4 mg baricitinib daily. Over half of the patients in the dose-escalation group achieved PGA 0 after 12 additional weeks of therapy. All outcome measures except Skindex-16 improved in this cohort, although not statistically significant due to the small sample size. Further evaluation with a larger cohort is needed to establish the safety and efficacy of this increased dosing, but our data suggest a dose-dependent response to baricitinib in LP.

Patient-reported QoL, in addition to pruritus and pain symptoms, improved with baricitinib. At baseline, the patients on trial had QoL and pruritus NRS scores analogous to poorly controlled, severe atopic dermatitis, as evidenced by mean overall Skindex-16 and pruritus NRS scores of 55.3 and 6.9, respectively (19, 23). By week 16, there were dramatic improvements in itch, with pruritus NRS decreasing from 6.9 (moderate-severe itch) to 1.2 (mild itch). NRS improvement from baseline occurred in 75% of patients (24).

The underlying mechanism of pruritus in LP remains unknown; however, similar responses were seen with topical JAK1/2 inhibition with topical ruxolitinib, implying that JAK1 and/or JAK2 play a central role in LP pruritus (16). We detected an increase in *IL13* mRNA expression in lesional LP skin, but not *IL4* in our bulk RNA-Seq data. This suggests that while Th2 responses are not prominent in LP, consistent with prior reports (25, 26), that Th2 cytokines, particularly IL-13, are likely present at low levels in inflamed LP skin and responsive to JAK inhibition and thereby contribute to the marked decrease in itch seen with baricitinib treatment (27).

The pathogenic antigen in LP is unknown, but it has been suggested that T cells are central disease mediators, with cytotoxicity mediated through IFN-γ priming of KCs through MHC class I induction and cell death (8). Consistent with those findings, the primary source of IFN-γ in LP is cytotoxic CD8^+^ and γ-Δ T cells. Here, we identify what we believe to be a novel subset of CXCL13^+^CD8^+^ T cells that are a major source of IFN-γ in LP and show oligoclonality, suggesting reactivity against a limited set of possible autoantigens. CXCL13^+^CD8^+^ T cells have been described as tumor-reactive cells triggered by immune-checkpoint blockade (28), but to our knowledge, this population has not previously been demonstrated to contribute to skin inflammation. CXCL13 regulates the tumor lymphocyte infiltrate, and CXCL13 expression on CD8^+^ T cells has been shown to be a predictor of immune checkpoint inhibitor response (29). Interestingly, lichenoid dermatitis is not an uncommon cutaneous side effect of immune-checkpoint inhibitor treatment (30), although the specific T cell lymphocyte population in that setting has not been previously explored. Notably, the 3 CD8^+^ T cell populations were localized at the dermal-epidermal junction and showed predicted interactions with basal layer KCs where enriched IFN responses and MHC class I and class II expression were observed. The restricted clonality of these cells, along with their cytotoxic features, suggest that these cells may be reacting against self-antigens in the basal layer of the epidermis with IFN-γ signaling priming basal cells toward cytotoxic attack, setting the stage for a vicious self-sustaining cycle of cytotoxic responses against self-antigens in basal KCs. Furthermore, the correlation between decreased frequency of CXCL13^+^CD8^+^ T cells with clinical improvement suggests that changes in this population may predict treatment response or a potential target of future treatments, which warrants further investigation. Literature-based network analysis of genes demonstrated this signaling to be dependent on JAK/STAT signaling (8). Consistent with this scenario, MHC class I expression rapidly decreases in basal KCs with baricitinib treatment. Taken these data together, inhibition of JAK2 with baricitinib protects KCs from IFN-γ–induced cytotoxic responses. In addition, MX1 was upregulated in our study and has been reported in LP. MX1/MXA and other chemokines, such as CXCL10, suggest potential additional roles for type I and III interferons in LP pathogenesis (31).

We did not observe evidence of involvement of IL-17 in LP pathogenesis, as previously suggested (32). However, most of the reports on IL-17 in LP have focused on oral LP, which was not included in our clinical trial. Our scRNA-Seq analysis has limitations, as we only had 1 patient with no improvement. There were also 2 patients who only had skin biopsy at week 0 or week 2.

Serious AEs did not occur with baricitinib. A single AE of neutropenia was deemed probably related to the study drug, yet this was mild and did not result in treatment discontinuation. Based on the promising results of this open-label, single-arm trial, future randomized controlled trials of baricitinib are warranted.

## Methods

### Sex as a biological variable.

Human skin samples from 1 male and 11 females were used in this study. Sex was not considered as a biological variable due to insufficient statistical power to analyze sex-stratified effects.

### Demographic reporting.

Demographic variables of race, ethnicity, and sex at birth (male or female) were defined by the investigators, and participants selected the classification they identified with. Racial and ethnic categories were defined in accordance with NIH guidelines.

### Trial design.

This single-arm, open-label, phase 2, first-in-human trial was conducted at Mayo Clinic, Arizona (ClinicalTrials.gov NCT05188521). Twelve patients with biopsy-proven cutaneous LP were administered 2 mg oral baricitinib once daily for 16 weeks. The primary endpoint was an overall response by PGA of skin at week 16, with treatment response defined as PGA 0 to 3 (with ≥ 50% score reduction, Supplemental Table 1). Secondary outcomes were changes in mCAILS, total body lesion count, affected BSA, pruritus NRS, pruritus VRS, pruritus VAS, pain NRS, and Skindex-16 (19, 20, 33–36). BSA was conducted using the hand method defined at 1% and the thumb at 0.1%. (37) Patients were evaluated at baseline (week 0) and weeks 2, 4, 8, 12, and 16. Treatment-responsive patients who did not achieve PGA grade 0 at week 16 were eligible to enroll in the dose escalation for an additional 12 weeks of treatment with 4 mg oral baricitinib daily. Complete responders at week 16 were reassessed at week 20, and partial responders at week 16 were reassessed at week 32, after an off-therapy period of 4 weeks, respectively.

Treatment efficacy, AEs, and QoL were assessed at each study visit. All lesions were annotated, photographed, and scored using the mCAILS criteria. The rationale for the use of mCAILS has been described previously (16). All clinical assessments were performed by the principal investigator and coinvestigators with questionable lesions scored by 2 investigators. The revised National Cancer Institute Common Terminology Criteria for Adverse Events, version 5.0, was used for AE reporting. Due to travel issues, one patient withdrew from the study after week 8. We used ITT analysis with the patient’s last observation to impute week 16 data.

Fresh-frozen tissue via 3 mm punch biopsies was collected at baseline and week 2 of LP lesional skin and normal -appearing skin for bulk RNA-Seq. Week 2 samples were designated as responsive (defined as a lesion with ≥50% response by mCAILS) or nonresponsive (defined as a lesion with <50% response by mCAILS) (33). Additional 6–8 mm biopsies of lesional tissue were taken at weeks 0 and 2 for spatial sequencing and scRNA-Seq. 5 mL blood samples were collected at both time points for scRNA-Seq of PBMCs. Standard photos were used at week 0 and week 2 for lesion identification; biopsies were taken at least 1 cm apart, and all biopsies were taken from the same lesion if possible or from the same body region.

### Eligibility criteria.

Patients aged 18 years or older with biopsy-proven cutaneous LP were eligible for the trial. Both treatment-naive and treatment-refractory disease were included. Key exclusion criteria included predominantly noncutaneous variants of LP (erosive, intertriginous, oral, facial, drug induced, vaginal), active infections, and other active inflammatory cutaneous conditions. See supplemental data for additional eligibility criteria (see Supplemental Eligibility Criteria).

### Tissue processing, transcriptomic processing, quality control, alignment, and bulk RNA-Seq analysis.

For bulk RNA-Seq, tissue was processed, RNA was isolated as previously described by our group (38), and 150bp paired-end reads were generated. The reads were adapter trimmed and aligned to the human genome hg38, with only the uniquely mapped reads used for expression level quantification. DESeq2 was used to perform read normalization and DE analyses.

### Generation of single-cell suspensions for scRNA-Seq.

Half of a 6 mm biopsy was cryopreserved in CryoStor CS10 media (BioLife Solutions). Samples were thawed on ice, washed briefly in cold HBSS (Gibco, Thermo Fisher Scientific) to remove residual CryoStor CS10 media, and bisected before being enzymatically digested in either 0.25% Trypsin-EDTA (Gibco, Thermo Fisher Scientific) with 10 U/mL DNase I (Thermo Fisher Scientific) for 1 hour at 37°C and quenched with FBS (Atlanta Biologicals) or 0.2% collagenase II (Life Technologies) and 0.2% collagenase V (Sigma) with 10U/ml DNase I in plain medium for 1.5 hours at 37°C with rotation. The resulting cell suspensions were filtered through 70 μm cell strainers twice and resuspended in PBS containing 0.04% BSA. Dermal and epidermal cells were combined in a 2:1 ratio. Cell suspensions from tissue and blood samples were submitted for scRNA-Seq, respectively; libraries were constructed by the University of Michigan Advanced Genomics Core on the 10X Genomics Chromium system with chemistry, version 2 and version 3, and sequenced on the Illumina NovaSeq 6000 sequencer to generate 150 bp paired-end reads.

### scRNA-Seq data analysis.

Data processing, including quality control, read alignment (hg38), and gene quantification, was conducted using the 10X Cell Ranger. The samples were then merged into a single expression matrix using the cellranger aggr pipeline. The R package Seurat (version 3.1.2) (39) was used to cluster the cells in the merged matrix. Cells with less than 500 transcripts or 100 genes or more than 10% of mitochondrial expression were first filtered out as low-quality cells. SoupX was utilized to remove ambient RNA reads. Doublets were detected and removed using scDblfinder (40, 41). The NormalizeData function was used to normalize the expression level for each cell with default parameters. The FindVariableFeatures function was used to select variable genes with default parameters. The FindIntegrationAnchors and IntegrateData functions were used to integrate the samples prepared using different 10X Chromium chemistries. Samples were batch corrected using Harmony, utilizing the donor as a batch. Subclustering was performed on the abundant T cell types. The FindClusters function in the Seurat R package was used to obtain the subclusters. Subclusters defined exclusively by mitochondrial gene expression, indicating low quality, were removed from further analysis. Subtypes were annotated by overlapping subcluster marker genes with canonical subtype signature genes. Using CellChat (version 2.1.2) (42), we used the default computeCommunProb trimean method, which approximates 25% truncated mean to calculate the average gene expression per cell group.

### Spatial sequencing analyses.

The spatial transcriptomic experiment was described in our previous work (43, 44). Briefly, the skin sample was frozen in OCT medium and stored at –80°C. SpaceRanger was utilized to map the reads to the custom hg19 genome with 18,517 lncRNA loci. The expression matrix was analyzed in Seurat. Spots expressing 200 or more genes, less than 25% mitochondrial reads, and less than 20% hemoglobin reads were kept. Normalization, scaling, and clustering were performed using Seurat. Cell type deconvolution was performed utilizing R package CARD, using the above scRNA-Seq data as a cell-type reference panel (45).

### T cell receptor clonality analyses.

10X Genomics software CellRanger count (version 7.0.1) was utilized to generate gene-expression data matrices within each cell. Fastq files were aligned to Hg38, 2020 version. 10X software CellRanger VDJ was utilized to map T cell receptors from V(D)J recombination within each sample matrix. Clonotypes of total CD8^+^ and CXCL13^+^CD8 T cells were tabulated using the V and J chains, and the most frequently identified clone was highlighted in a pie chart. Cell-type annotations were applied to cell barcodes within Seurat utilizing a custom marker gene list. Subsequently, cell-type annotations were reapplied to corresponding cell barcodes flagged by CellRanger VDJ.

### IHC.

Frozen tissue sections were dried at room temperature for 30 minutes. Slides were fixed by acetone (stored at –20°C) for 10 minutes and then treated with 3% H_2_O_2_ (5 minutes) and blocked using 10% secondary source serum (30 minutes). Overnight incubation (4°C) was then performed using anti-human antibodies against CXCL10 (Thermo Fisher Scientific, catalog 701225), CXCL9 (R&D Systems, catalog AF392) pSTAT2 (Thermo Fisher Scientific, catalog BS-3428R), CD3 (Abcam, catalog AB135372), and IFN-γ (Abcam, catalog AB25101). Slides were washed and treated with secondary antibody, peroxidase (30 minutes), and diaminobenzidine substrate.

### Statistics.

Patient demographics, clinical characteristics, and outcomes were summarized as mean, SD, median, interquartile range for continuous variables, and frequency and percentages for categorical variables. Primary and secondary outcome differences between baseline and week 16 were compared using Wilcoxon’s signed rank test for continuous variables and McNemar’s test for binary variables. One patient withdrew from the study after week 8, and this patient’s last observation (week 8) was used to impute their week 16 data in the ITT analysis. Exact binomial method was used to calculate the treatment-response rate at week 16 and its corresponding 95% confidence interval. All analyses were conducted with R, version 4.1.2 (R Foundation for Statistical Computing). All the tests were 2-sided and a *P* value of less than 0.05 was considered statistically significant.

### Study approval.

The Mayo Clinic Institutional Review Board approved the study (IRB 21-003075), and all patients provided written, informed consent prior to participation. All patients provided written, informed consent for photographs, and the record of informed consent has been retained.

### Data availability.

The RNA-Seq data discussed in this publication is available in the NCBI’s Gene Expression Omnibus database (GEO GSE280583 [bulk RNA] and GSE280584 [scRNA]). Values for all data points in graphs are reported in the Supporting Data Values file.

## Author contributions

Study conceptualization and design were contributed by ARM and JEG. Protocol writing was contributed by ARM, CB, and MP. Data collection was completed by ARM, SZ, and EB. Statistical analysis was done by NZ and XL. Tissue processing and transcriptomic analysis were contributed by AH, XL, RB, JF, and TD. IHC staining and analysis were done by XX, OP, and LCT. Original manuscript writing was contributed by ASH, JAK, ARM, and JEG. Manuscript review and editing was done by ARM, JEG, MRP, JMK, TD, ACB, MGK, DJD, MLY, SN, ALS, ZLR, EO, PB, BB, JAK, and ASH. Authorship order for co–first authors was determined by alphabetical order.

## Supplementary Material

Supplemental data

ICMJE disclosure forms

Supplemental table 5

Supporting data values

## Figures and Tables

**Table 3:**
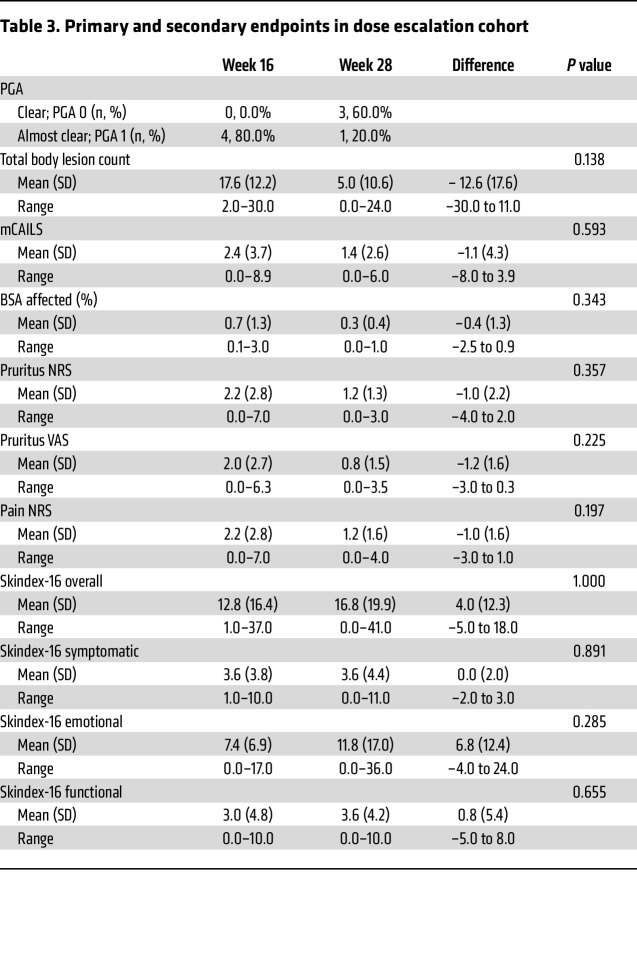
Primary and secondary endpoints in dose escalation cohort

**Table 2:**
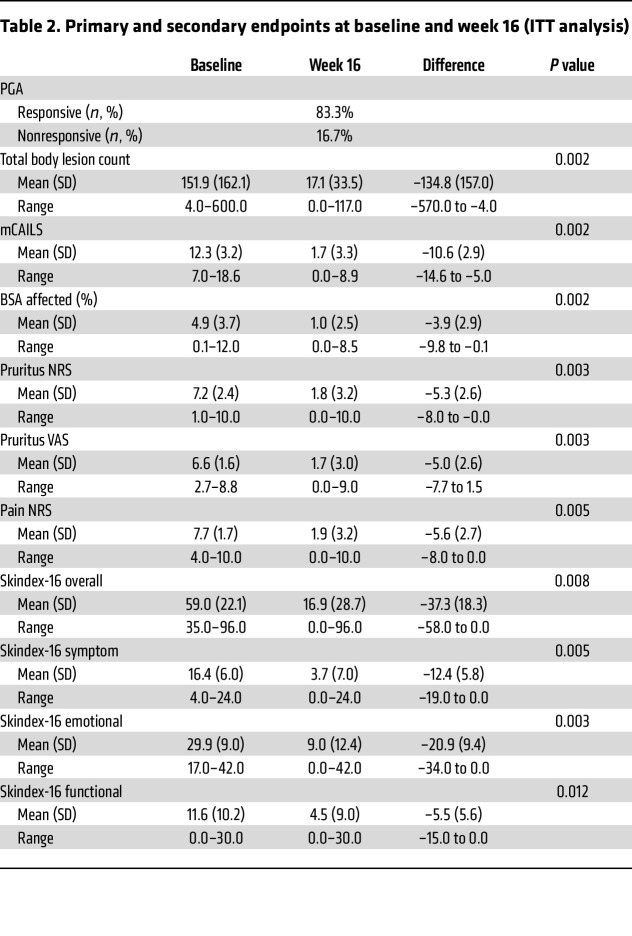
Primary and secondary endpoints at baseline and week 16 (ITT analysis)

**Table 1:**
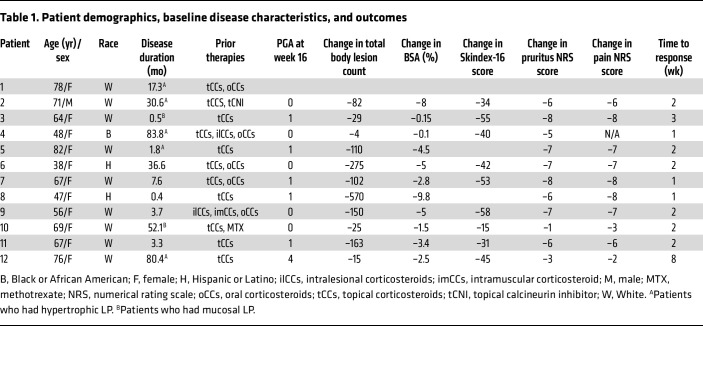
Patient demographics, baseline disease characteristics, and outcomes

## Version 1 11/14/2024

In-Press Preview

## Version 2 01/16/2025

Electronic publication

## References

[2] Boyd AS, Neldner KH (1991). Lichen planus. J Am Acad Dermatol.

[3] Boch K (2021). Lichen planus. Front Med (Lausanne).

[4] Gorouhi F (2014). Cutaneous and mucosal lichen planus: a comprehensive review of clinical subtypes, risk factors, diagnosis, and prognosis. ScientificWorldJournal.

[5] Pietschke K (2021). The inflammation in cutaneous lichen planus is dominated by IFN-γ and IL-21-A basis for therapeutic JAK1 inhibition. Exp Dermatol.

[6] Groves RW (1993). Vascular cell adhesion molecule-1: expression in normal and diseased skin and regulation in vivo by interferon gamma. J Am Acad Dermatol.

[7] Bennion SD (1995). In three types of interface dermatitis, different patterns of expression of intercellular adhesion molecule-1 (ICAM-1) indicate different triggers of disease. J Invest Dermatol.

[8] Shao S (2019). IFN-γ enhances cell-mediated cytotoxicity against keratinocytes via JAK2/STAT1 in lichen planus. Sci Transl Med.

[9] Szabo SJ (2002). Distinct effects of T-bet in TH1 lineage commitment and IFN-gamma production in CD4 and CD8 T cells. Science.

[10] Damsky W (2020). Treatment of severe lichen planus with the JAK inhibitor tofacitinib. J Allergy Clin Immunol.

[11] Yang CC (2018). Tofacitinib for the treatment of lichen planopilaris: a case series. Dermatol Ther.

[12] Moussa A (2022). Effective treatment of oral lichen planus with the JAK inhibitor baricitinib. Australas J Dermatol.

[13] Balestri R (2022). Treatment of oral erosive lichen planus with upadacitinib. JAMA Dermatol.

[14] Kooybaran NR (2021). Alleviation of erosive oral and esophageal lichen planus by the JAK1 inhibitor upadacitinib. J Dtsch Dermatol Ges.

[15] Seiringer P (2020). Tofacitinib in hypertrophic lichen planus. Acta Derm Venereol.

[16] Brumfiel CM (2022). Ruxolitinib cream in the treatment of cutaneous lichen planus: a prospective, open-label study. J Invest Dermatol.

[17] Pünchera J, Laffitte E (2022). Treatment of severe nail lichen planus with baricitinib. JAMA Dermatol.

[18] Moussa A (2022). Treatment of lichen planopilaris with baricitinib: a retrospective study. J Am Acad Dermatol.

[19] Chren MM (2012). The Skindex instruments to measure the effects of skin disease on quality of life. Dermatol Clin.

[20] Chren MM (2001). Measurement properties of Skindex-16: a brief quality-of-life measure for patients with skin diseases. J Cutan Med Surg.

[21] Nath N (2010). Modulation of stress genes expression profile by nitric oxide-releasing aspirin in Jurkat T leukemia cells. Biochem Pharmacol.

[22] Liu X (2019). Genome-wide analysis identifies NR4A1 as a key mediator of T cell dysfunction. Nature.

[23] Simpson EL (2016). Two phase 3 trials of dupilumab versus placebo in atopic dermatitis. N Engl J Med.

[24] Yosipovitch G (2019). Peak Pruritus Numerical Rating Scale: psychometric validation and responder definition for assessing itch in moderate-to-severe atopic dermatitis. Br J Dermatol.

[25] Soumelis V (2002). Human epithelial cells trigger dendritic cell mediated allergic inflammation by producing TSLP. Nat Immunol.

[26] Storan ER (2015). Role of cytokines and chemokines in itch. Handb Exp Pharmacol.

[27] Oetjen LK (2017). Sensory neurons co-opt classical immune signaling pathways to mediate chronic itch. Cell.

[28] Liu B (2022). Single-cell meta-analyses reveal responses of tumor-reactive CXCL13^+^ T cells to immune-checkpoint blockade. Nat Cancer.

[29] Litchfield K (2021). Meta-analysis of tumor- and T cell-intrinsic mechanisms of sensitization to checkpoint inhibition. Cell.

[30] Schaberg KB (2016). Immunohistochemical analysis of lichenoid reactions in patients treated with anti-PD-L1 and anti-PD-1 therapy. J Cutan Pathol.

[31] Wenzel J, Tuting T (2008). An IFN-associated cytotoxic cellular immune response against viral, self-, or tumor antigens is a common pathogenetic feature in “interface dermatitis”. J Invest Dermatol.

[32] Husein-ElAhmed H, Steinhoff M (2022). Potential role of INTERLEUKIN-17 in the pathogenesis of oral lichen planus: a systematic review with META-analysis. J Eur Acad Dermatol Venereol.

[33] Olsen EA (2011). Clinical end points and response criteria in mycosis fungoides and Sézary syndrome: a consensus statement of the International Society for Cutaneous Lymphomas, the United States Cutaneous Lymphoma Consortium, and the Cutaneous Lymphoma Task Force of the European Organisation for Research and Treatment of Cancer. J Clin Oncol.

[34] Duvic M (2001). Phase 2 and 3 clinical trial of oral bexarotene (Targretin capsules) for the treatment of refractory or persistent early-stage cutaneous T-cell lymphoma. Arch Dermatol.

[35] Phan NQ (2012). Assessment of pruritus intensity: prospective study on validity and reliability of the visual analogue scale, numerical rating scale and verbal rating scale in 471 patients with chronic pruritus. Acta Derm Venereol.

[36] Reich A (2012). Visual analogue scale: evaluation of the instrument for the assessment of pruritus. Acta Derm Venereol.

[37] Kim BS (2020). Treatment of atopic dermatitis with ruxolitinib cream (JAK1/JAK2 inhibitor) or triamcinolone cream. J Allergy Clin Immunol.

[38] Tsoi LC (2019). Atopic dermatitis Is an IL-13-dominant disease with greater molecular heterogeneity compared to psoriasis. J Invest Dermatol.

[39] Butler A (2018). Integrating single-cell transcriptomic data across different conditions, technologies, and species. Nat Biotechnol.

[40] Young MD, Behjati S (2020). SoupX removes ambient RNA contamination from droplet-based single-cell RNA sequencing data. Gigascience.

[41] Germain PL (2021). Doublet identification in single-cell sequencing data using scDblFinder. F1000Res.

[42] Jin S (2021). Inference and analysis of cell-cell communication using CellChat. Nat Commun.

[43] Ma F (2023). Single cell and spatial sequencing define processes by which keratinocytes and fibroblasts amplify inflammatory responses in psoriasis. Nat Commun.

[44] Ma F (2024). Systems-based identification of the Hippo pathway for promoting fibrotic mesenchymal differentiation in systemic sclerosis. Nat Commun.

[45] Ma Y, Zhou X (2022). Spatially informed cell-type deconvolution for spatial transcriptomics. Nat Biotechnol.

