# Supplementary material for: Rapid response of lichen planus to baricitinib associated with suppression of cytotoxic CXCL13+CD8+ T cells
Source: J Clin Invest. 2024 Nov 14;135(2):e179436. doi: 10.1172/JCI179436 (PMC11735091; doi:10.1172/JCI179436)
Supplement: Supplemental data [file jci-135-179436-s025.pdf]

Supplemental Table 1. Physician Global Assessment (intention-to-treat analysis)

PGA	Week 1 (n=12)	Week 2 (n=12)	Week 3 (n=12)	Week 4 (n=12)	Week 8 (n=12)	Week 12 (n=12)	Week 16 (n=11)	Week 20 (n=11)
**Missing**	4	0	4	0	0	1	0	0
**Grade 0**	0 (0%)	0 (0%)	0 (0%)	0 (0%)	6 (50.0%)	5 (45.5%)	5 (41.7%)	4 (33..3%)
**Grade 1**	0 (0%)	1 (8.3%)	2 (25.0%)	4 (33.3%)	1 (8.3%)	4 (36.4%)	5 (41.7%)	5 (41.7%)
**Grade 2**	0 (0%)	3 (25.0%)	2 (25.0%)	3 (25.0%)	1 (8.3%)	0 (0%)	0 (0%)	1 (8.3%)
**Grade 3**	3 (37.5%)	5 (41.7%)	3 (37.5%)	2 (16.7%)	2 (16.7%)	2 (18.2%)	0 (0%)	1 (8.3%)
**Grade 4**	3 (37.5%)	2 (16.7%)	1 (12.5%)	3 (25.0%)	2 (16.7%)	0 (0%)	1 (8.3%)	0 (0%)
**Grade 5**	2 (25.0%)	1 (8.3%)	0 (0%)	0 (0%)	0 (0%)	0 (0%)	0 (0%)	0 (0%)

Grade 0: Completely clear, no evidence of disease (100% improvement)

Grade 1: Almost clear, very significant clearance (>=90% to <100%)

Grade 2: Marked improvement, significant improvement (>=75% to <90%)

Grade 3: Moderate improvement, intermediate between slight and marked (>=50% to <75%)

Grade 4: Slight improvement, some improvement (>=25% to <50%) however, significant evidence of disease remains

Grade 5: No change, disease has not changed from baseline condition (+/- <25%)

Supplemental Table 2. Primary and secondary endpoints at baseline and week 16 (per-protocol analysis)

	**Baseline**	**Week 16**	**Difference**	***P* value**
**PGA**				
Responsive (n, %)		90.9%		
Non-responsive (n, %)		9.1%		
**Total Body Lesion Count**				0.003
Mean (SD)	146.6 (168.9)	8.0 (12.0)	-138.6 (164.0)	
Range	4.0 – 600.0	0.0 – 30.0	-570.0 to -4.0	
**mCAILS**				0.003
Mean (SD)	11.8 (2.7)	1.1 (2.7)	-10.6 (3.1)	
Range	7.0 – 14.6	0.0 – 8.9	-14.6 to -5.0	
**BSA affected (%)**				0.003
Mean (SD)	4.2 (3.1)	0.3 (0.9)	-3.9 (3.0)	
Range	0.1 – 10.0	0.0 – 3.0	-9.8 to -0.1	
**Pruritus NRS**				0.003
Mean (SD)	6.9 (2.4)	1.1 (2.1)	-5.8 (2.1)	
Range	1.0 – 10.0	0.0 – 7.0	-8.0 to -1.0	
**Pruritus VAS**				0.003
Mean (SD)	6.6 (1.7)	1.0 (2.0)	-5.6 (1.7)	
Range	2.7 – 8.8	0.0 – 6.3	-7.7 to 2.5	
**Pain NRS**				0.005
Mean (SD)	7.5 (1.6)	1.2 (2.0)	-6.2 (2.1)	
Range	4.0 – 10.0	0.0 – 7.0	-8.0 to 2.0	
**Skindex-16 overall**				0.008
Mean (SD)	55.3 (19.4)	9.0 (12.2)	-41.4 (13.6)	
Range	35.0 – 90.0	0.0 – 37.0	-58.0 to -15.0	
**Skindex-16 symptom**				0.005
Mean (SD)	15.6 (5.7)	1.8 (3.0)	-13.6 (4.4)	
Range	4.0 – 24.0	0.0 – 10.0	-19.0 to -4.0	
**Skindex-16 emotional**				0.003
Mean (SD)	28.8 (8.6)	6.0 (7.0)	-22.8 (7.0)	
Range	17.0 – 42.0	0.0 – 17.0	-34.0 to 10.0	
**Skindex-16 functional**				0.012
Mean (SD)	9.9 (8.8)	2.0 (3.4)	-6.0 (5.6)	
Range	0.0 – 29.0	0.0 – 10.0	-15.0 to 0.0	

Supplemental Table 3. Physician Global Assessment in dose escalation cohort

PGA	Week 16 (n=5)	Week 20 (n=5)	Week 24 (n=5)	Week 28 (n=5)	Week 32 (n=5)
**Grade 0**	0 (0%)	1 (20.0%)	1 (20.0%)	3 (60.0%)	1 (20.0%)
**Grade 1**	4 (80.0%)	3 (60.0%)	2 (40.0%)	1 (20.0%)	1 (20.0%)
**Grade 2**	0 (0%)	0 (0%)	0 (0%)	0 (0%)	0 (0%)
**Grade 3**	0 (0%)	1 (20.0%)	1 (20.0%)	0 (0%)	2 (40.0%)
**Grade 4**	1 (20.0%)	0 (0%)	0 (0%)	1 (20.0%)	0 (0%)
**Grade 5**	0 (0%)	0 (0%)	1 (20.0%)	0 (0%)	1 (20.0%)

Supplemental Table 4. Adverse Events

	Possible/Probable/Definite	Unrelated/Unlikely	Total
**Adverse Event**			
Ankle cramp	0 (0.0%)	1 (9.1%)	1 (8.3%)
Blood cell count decreased	1 (100.0%)	0 (0.0%)	1 (8.3%)
Chest tenderness	0 (0.0%)	1 (9.1%)	1 (8.3%)
COVID	0 (0.0%)	1 (9.1%)	1 (8.3%)
Lichen planus flare	0 (0.0%)	1 (9.1%)	1 (8.3%)
Migraine with pain above right eye to the cheek	0 (0.0%)	1 (9.1%)	1 (8.3%)
Night sweats	0 (0.0%)	1 (9.1%)	1 (8.3%)
Pain lower right leg and right side	0 (0.0%)	1 (9.1%)	1 (8.3%)
Pain upper right side	0 (0.0%)	1 (9.1%)	1 (8.3%)
Shortness of breath, chest pain	0 (0.0%)	1 (9.1%)	1 (8.3%)
Upper respiratory tract infection/nasopharyngitis (common cold)	0 (0.0%)	1 (9.1%)	1 (8.3%)
Visual field changes, floaters and intermittent flashing left eye	0 (0.0%)	1 (9.1%)	1 (8.3%)
**Is the adverse event serious**			
Missing	0	1	1
No	1 (100.0%)	10 (100.0%)	11 (100.0%)
Yes	0 (0.0%)	0 (0.0%)	0 (0.0%)
**Severity Grade**			
Mild	1 (100.0%)	6 (54.5%)	7 (58.3%)
Moderate	0 (0.0%)	3 (27.3%)	3 (25.0%)
Severe	0 (0.0%)	2 (18.2%)	2 (16.7%)

Supplemental Table 5. DEGs in lesional LP vs non-lesional skin. *This large table has been uploaded as a separate spreadsheet.*

Supplemental Table 6. Quality metrics for scRNA-seq from lesional LP skin with Week 16 Physician Global Assessment (PGA) score.

Donor ID	nCell	nGene	nUMI	PGA Week 16 (Score)
3	1536	1923.476	5503.848	1
5	2832	1414.347	3672.448	1
6	2753	1700.903	4653.576	0
7	6880	885.8241	2095.372	1
8	5080	1897.117	5700.216	1
9	3835	2244.022	7164.77	0
10	1723	1959.364	6864.3	0
11	647	277.7635	405.9384	1
12	5539	2144.6	5916.644	4

Supplemental Table 7. Quality metrics for scRNA-seq from peripheral blood mononuclear cells (PBMCs) with Week 16 Physician Global Assessment (PGA) score.

Donor ID	nCell	nGene	nUMI	PGA Week 16 Score
1	17246	1812	5225	*
2	25984	1924	5371.7	0
3	26091	1991	5724	1
4	26337	2079	5989	0
5	23808	1979	5485	1
6	21288	1718	4991	0
7	14467	1417	3735.7	1
10	11625	1801	4543	0
11	11281	1804	4530	1
12	12834	1618	3913.7	4

*Patient withdrew from study after week 8, as described in Methods section

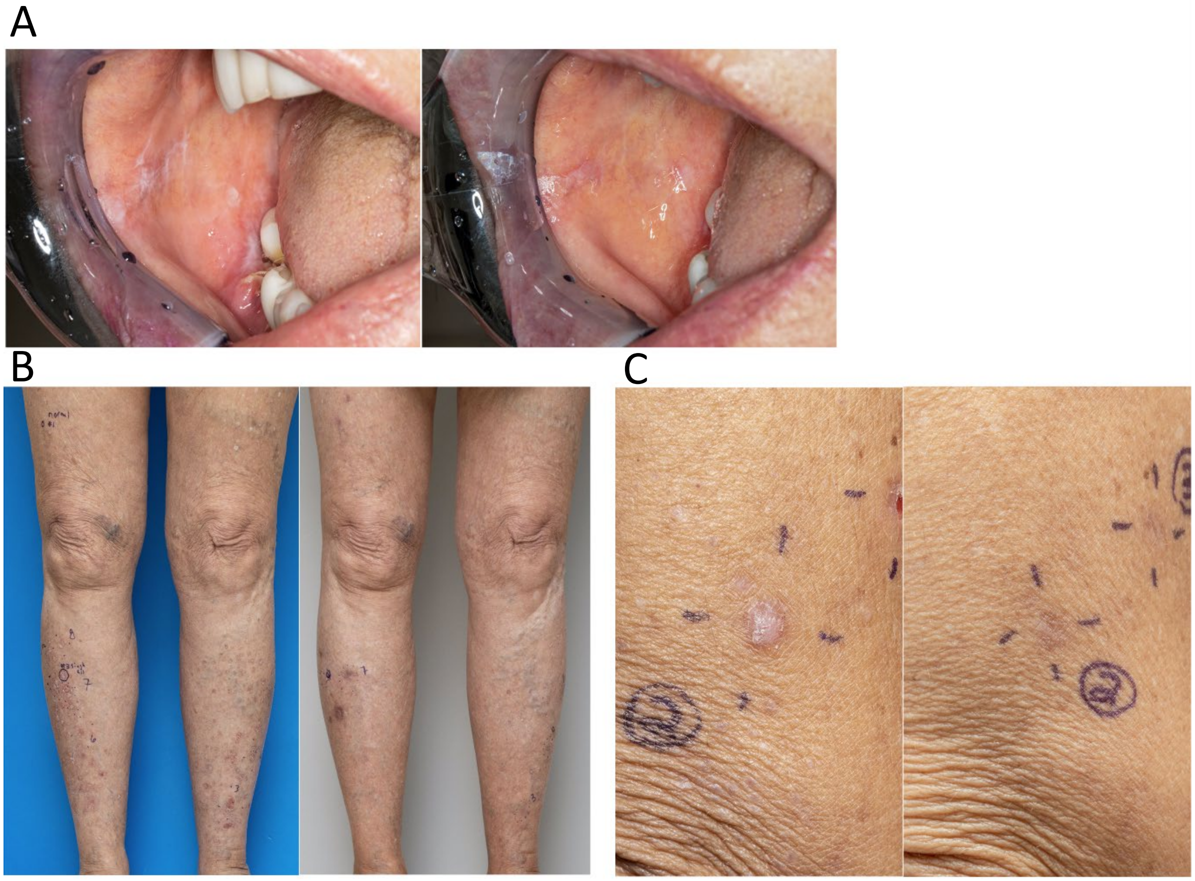

Supplemental Figure 1. Example images of LP response to baricitinib. **(A)** Mucosal response to baricitinib (Week 0 vs. Week 16). **(B)** Cutaneous response to baricitinib (Week 0 vs Week 20). **(C)** Cutaneous response to baricitinib (Week 0 vs Week 16).

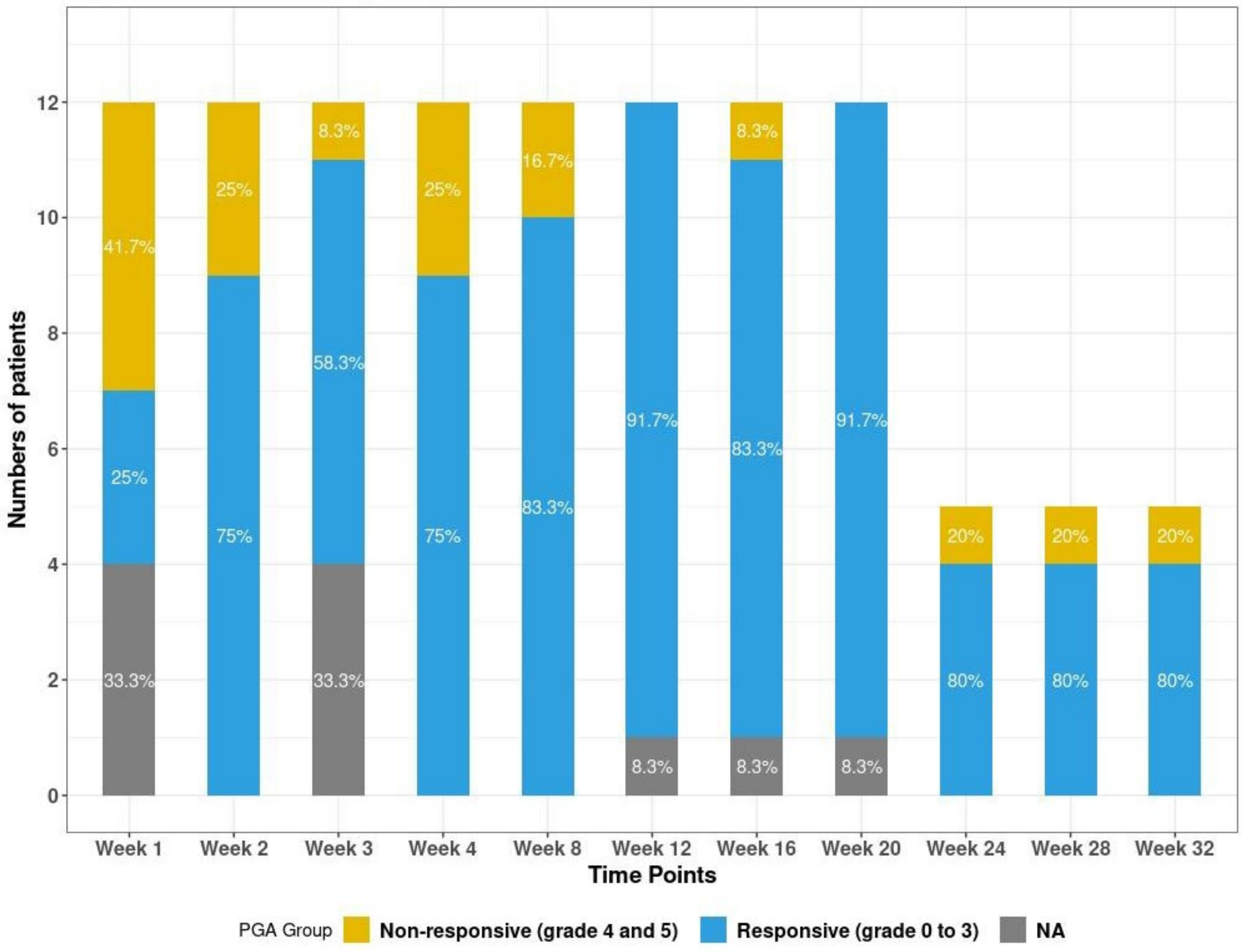

Supplemental Figure 2. Physician Global Assessment (PGA) percentage by timepoints. Treatment response rates as defined by PGA from baseline to week 16.

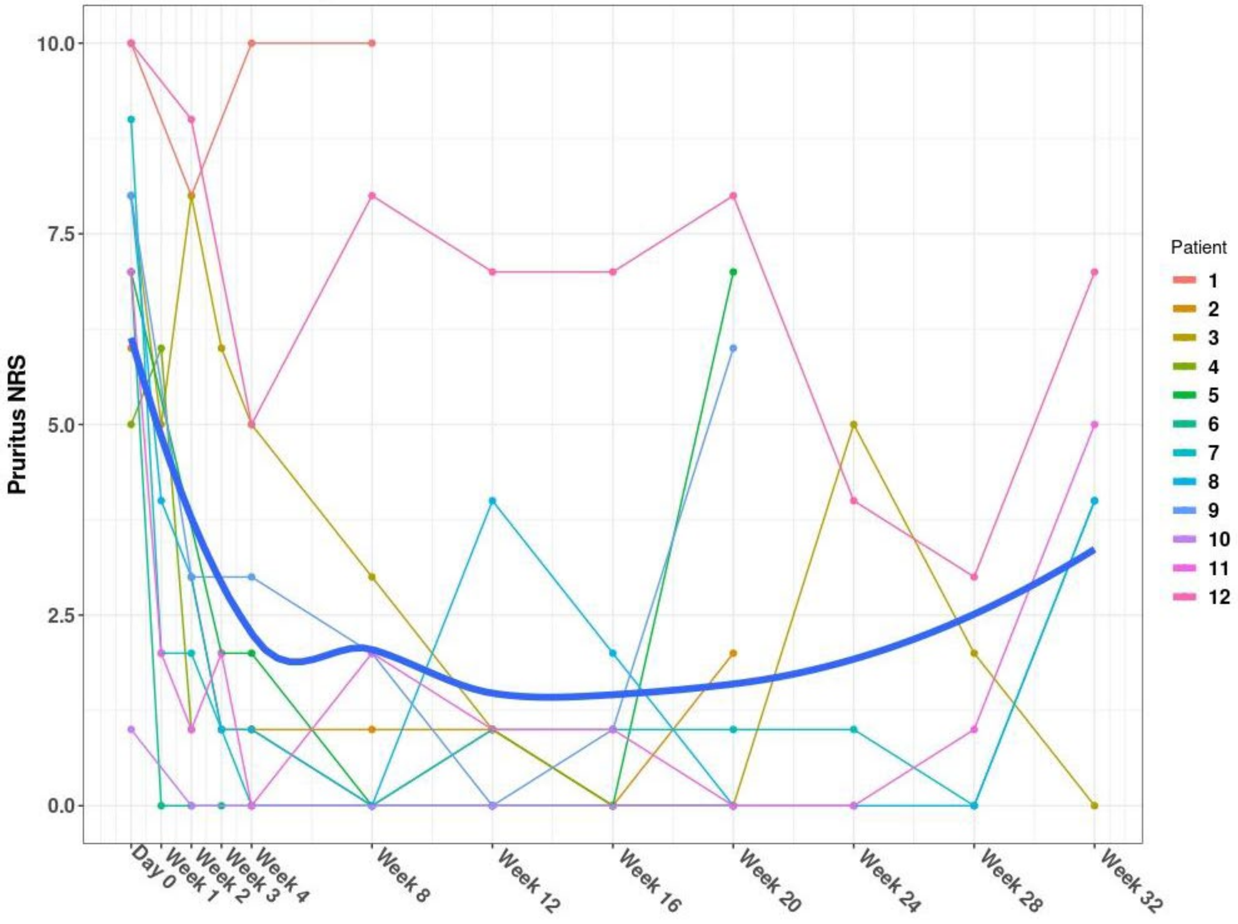

Supplemental Figure 3. Pruritus NRS by time for each participant from baseline to week 32.

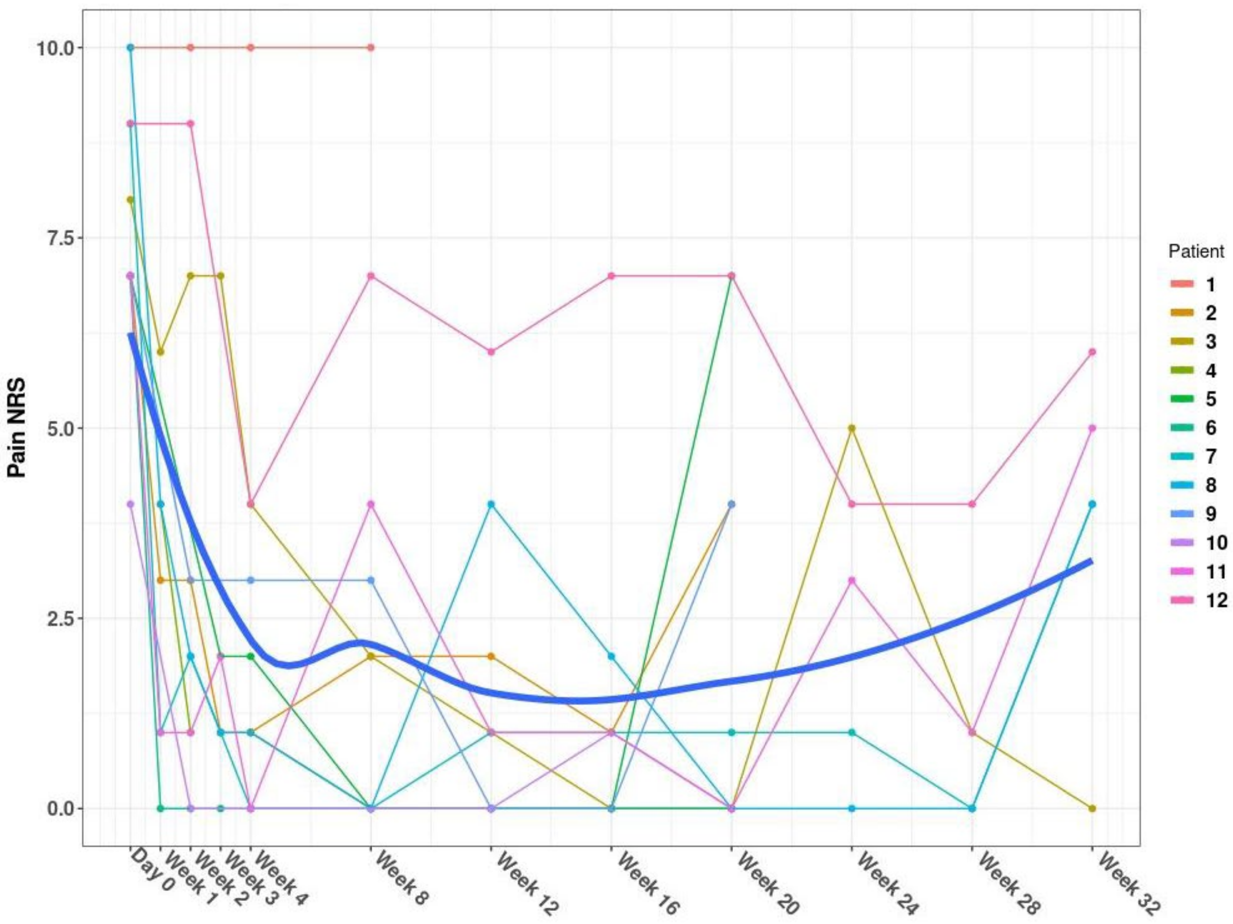

Supplemental Figure 4. Pain NRS by time for each participant from baseline to week 32.

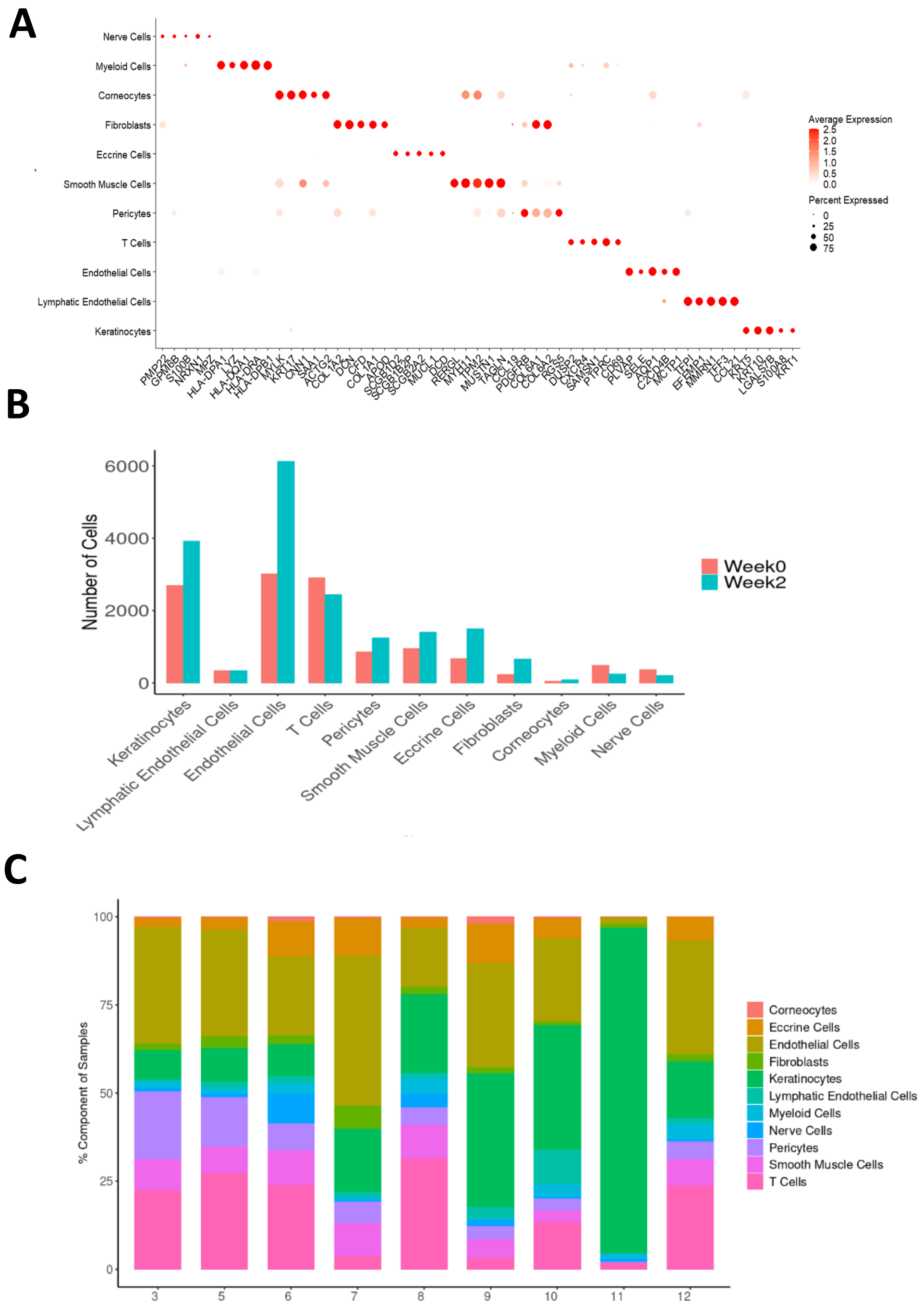

Supplemental Figure 5. (A) Top 5 gene markers in major cell types in lesional LP skin. **(B)** Cell numbers at baseline (week 0, red) and week 2 (blue) (n=9). **(C)** Proportion of cell type based on donor ID

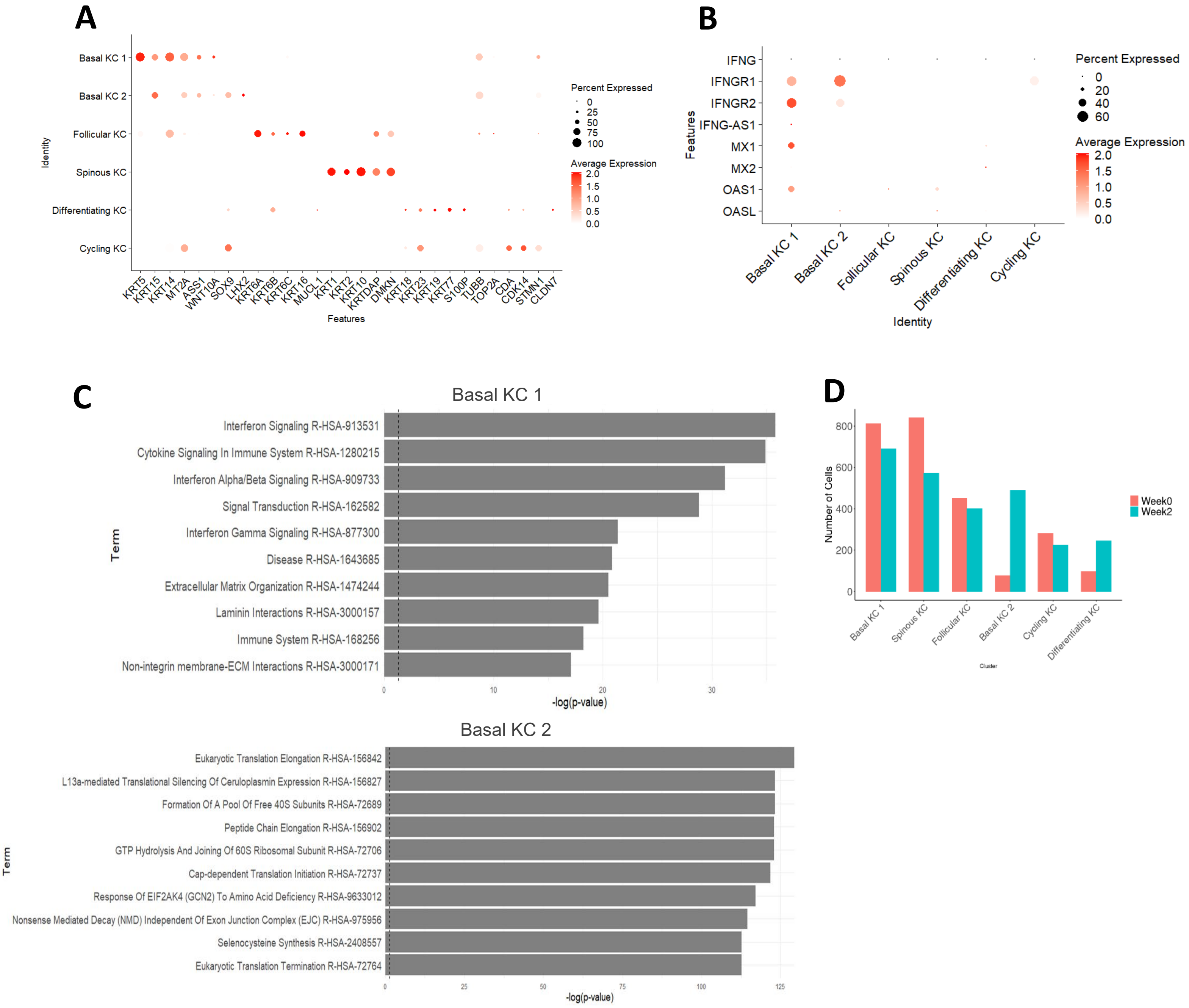

Supplemental Figure 6. (A) Top 5 keratinocyte markers for each keratinocyte subset identified in lesional LP skin. **(B)** Type II IFN and IFN-signature genes in different KC subsets. **(C)** Enriched Gene-Ontology pathways in "basal KC 1" and "basal KC 2" subsets compared to other keratinocytes. **(D)** Total cell numbers in each keratinocyte subsets in baseline (week 0, red) and week 2 (blue) (n=9).

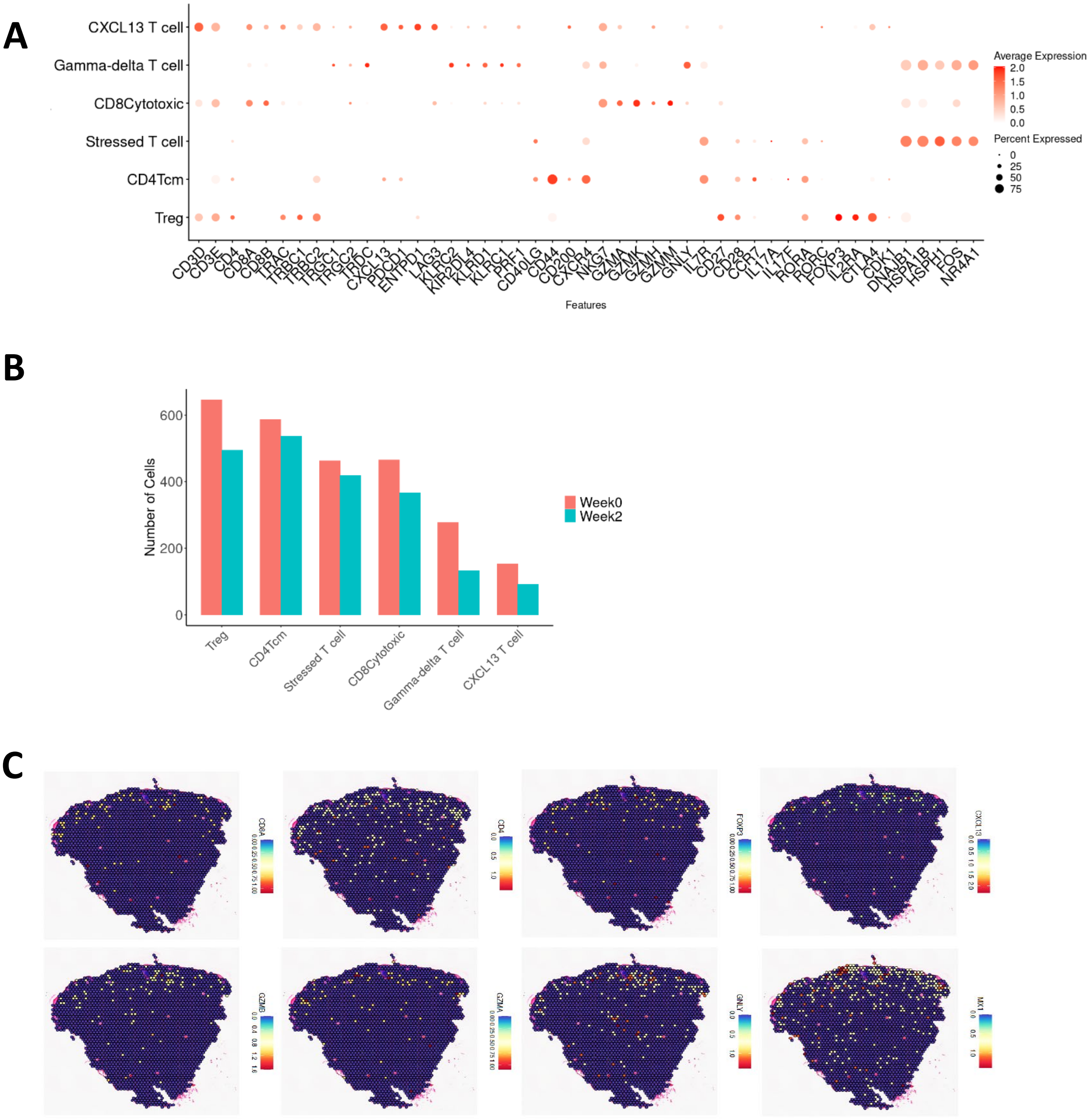

Supplemental Figure 7. (A) Marker genes for T cell subsets identified in lesional LP skin. **(B)** T cell numbers at baseline (red) and at week 2 after baricitinib treatment (n=9)**. (C)** Spatial deconvolution of T-cell subsets in lesional LP samples (representative of n=9).

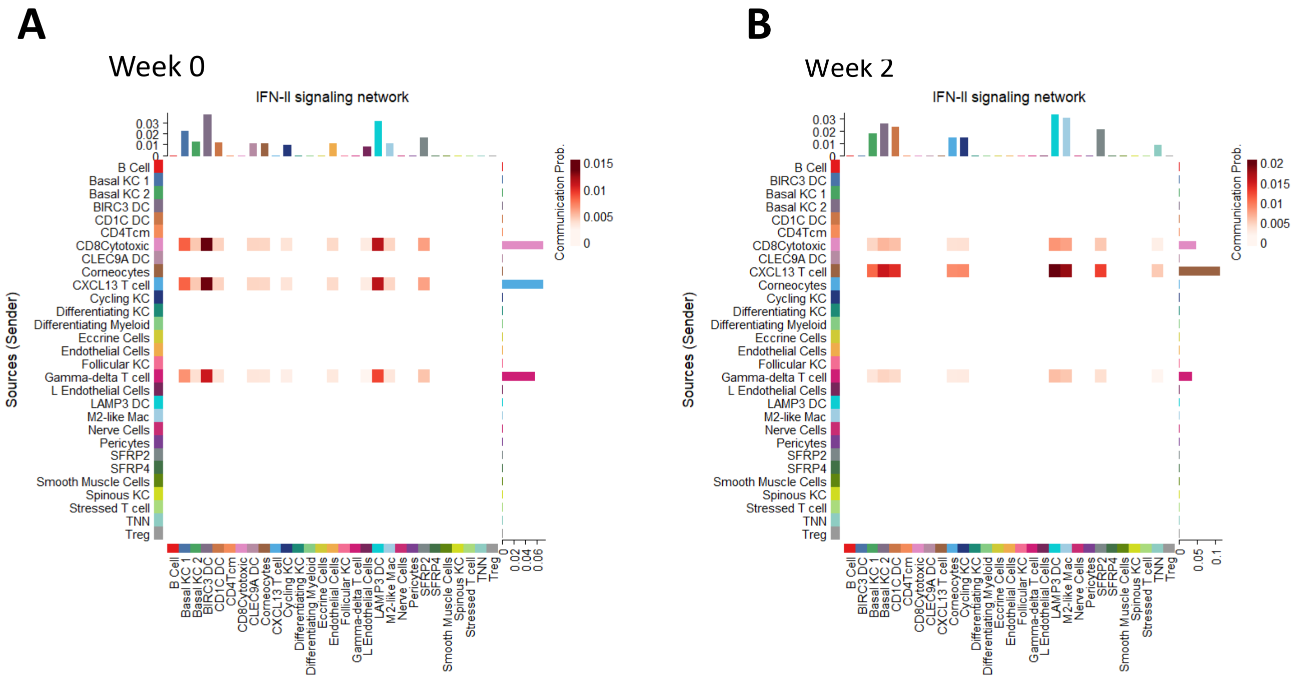

Supplemental Figure 8. (A) Heatmap showing the predicted interaction probability in the cell-cell communication network in Week. **(B)** Week 2 in the IFN-II signaling network.

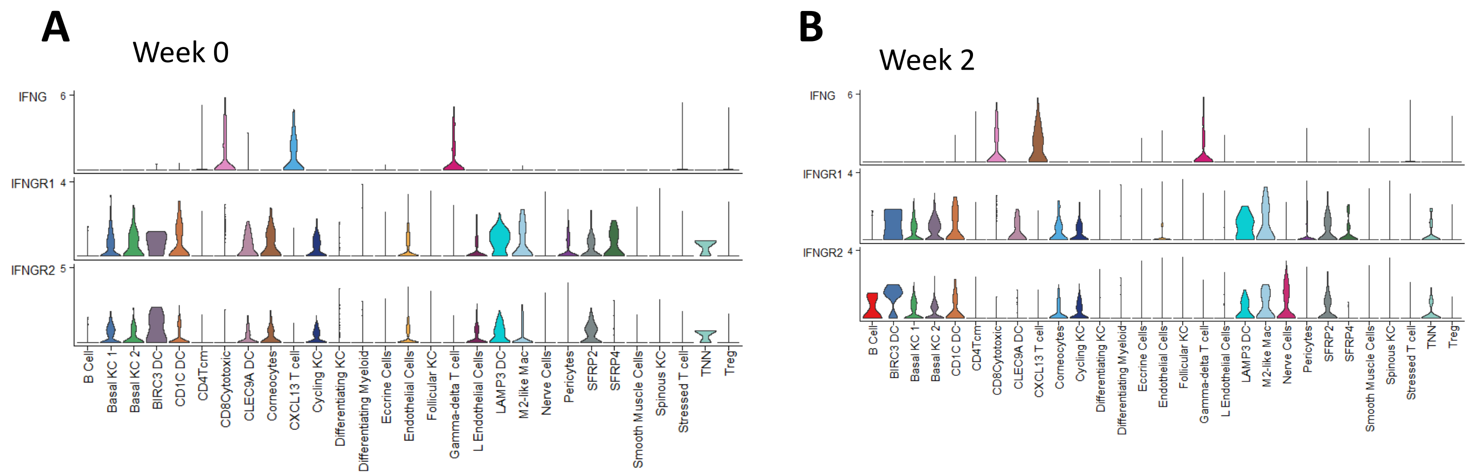

Supplemental Figure 9. (A) Expression of IFN-II signaling-related genes such as IFNG, IFNGR1, and IFNGR2 in Week 0. **(B)** Expression of IFN-II signaling-related genes such as IFNG, IFNGR1, and IFNGR2 in Week 2.

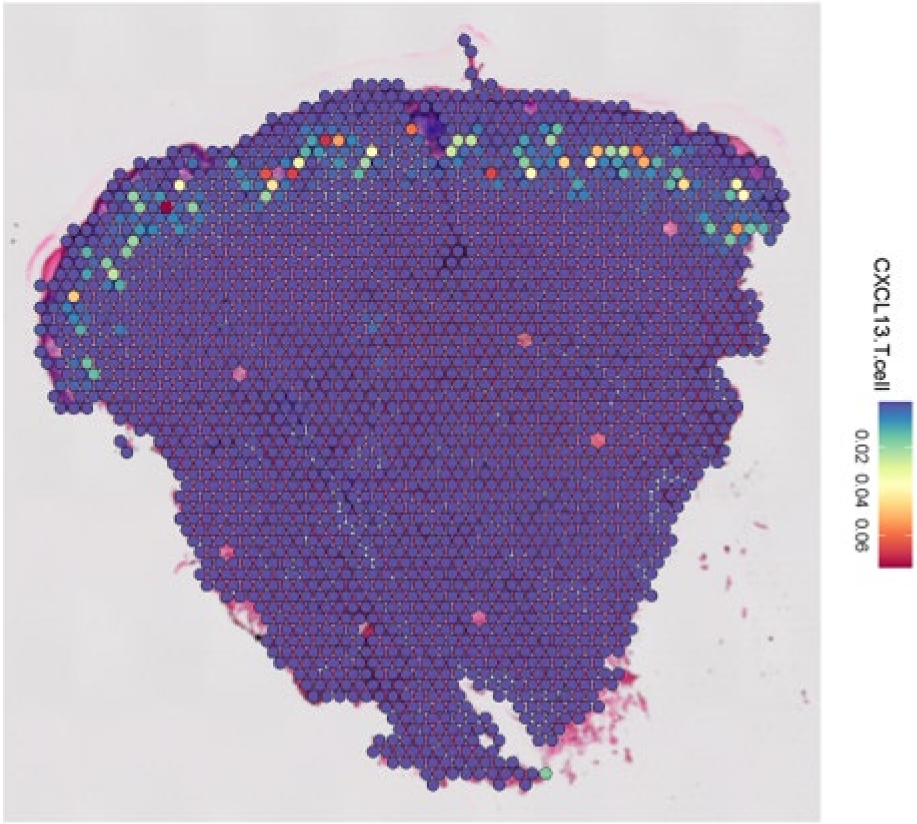

Supplemental Figure 10. Spatial expression of CXCL 13 T-cells in a representative lesional skin sample

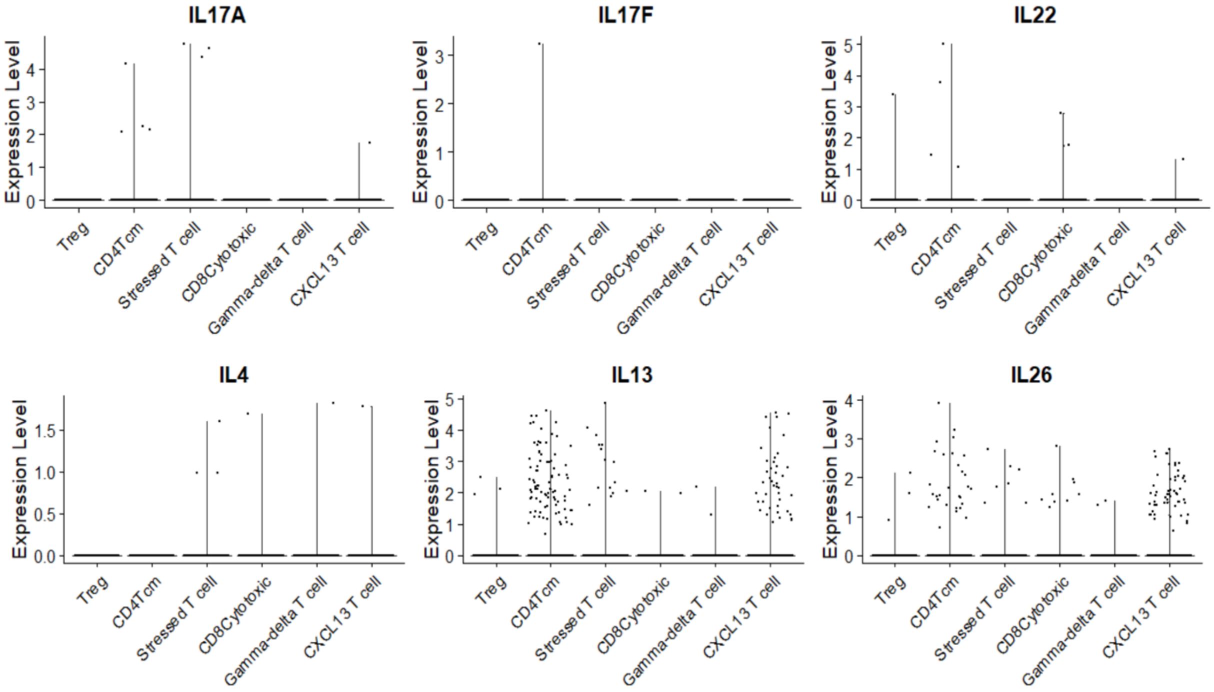

Supplemental Figure 11. Expanded panel of cytokine expression in lesional LP T-cells.

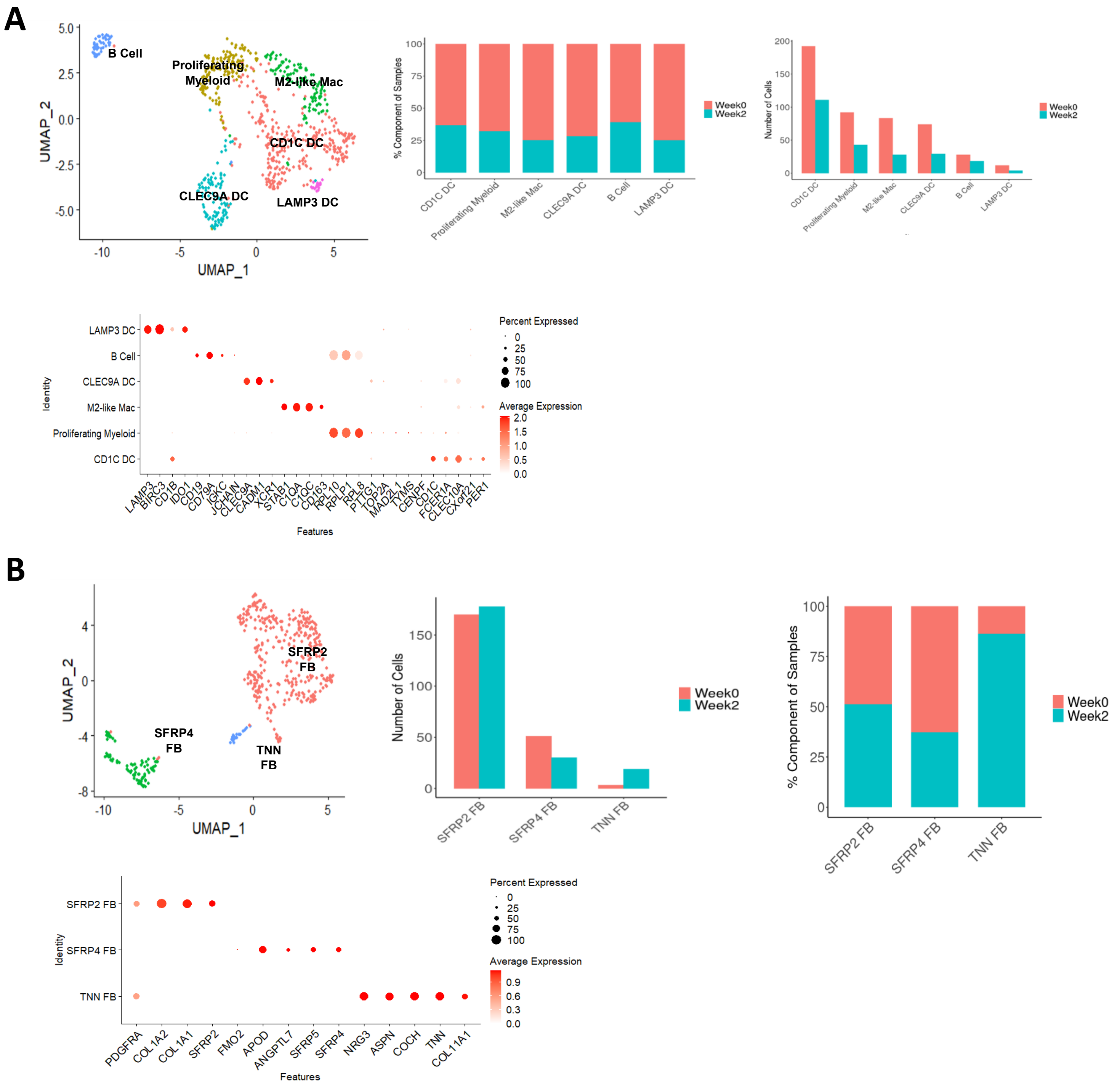

Supplemental Figure 12. (A) Myeloid and B cell populations in lesional LP skin. **(B)** Fibroblast subpopulations in LP skin. Data are shown for week 0 (Red) and week 2 (blue), along with the top 4 marker genes (n=10 at week 0 and n=10 at week 2).

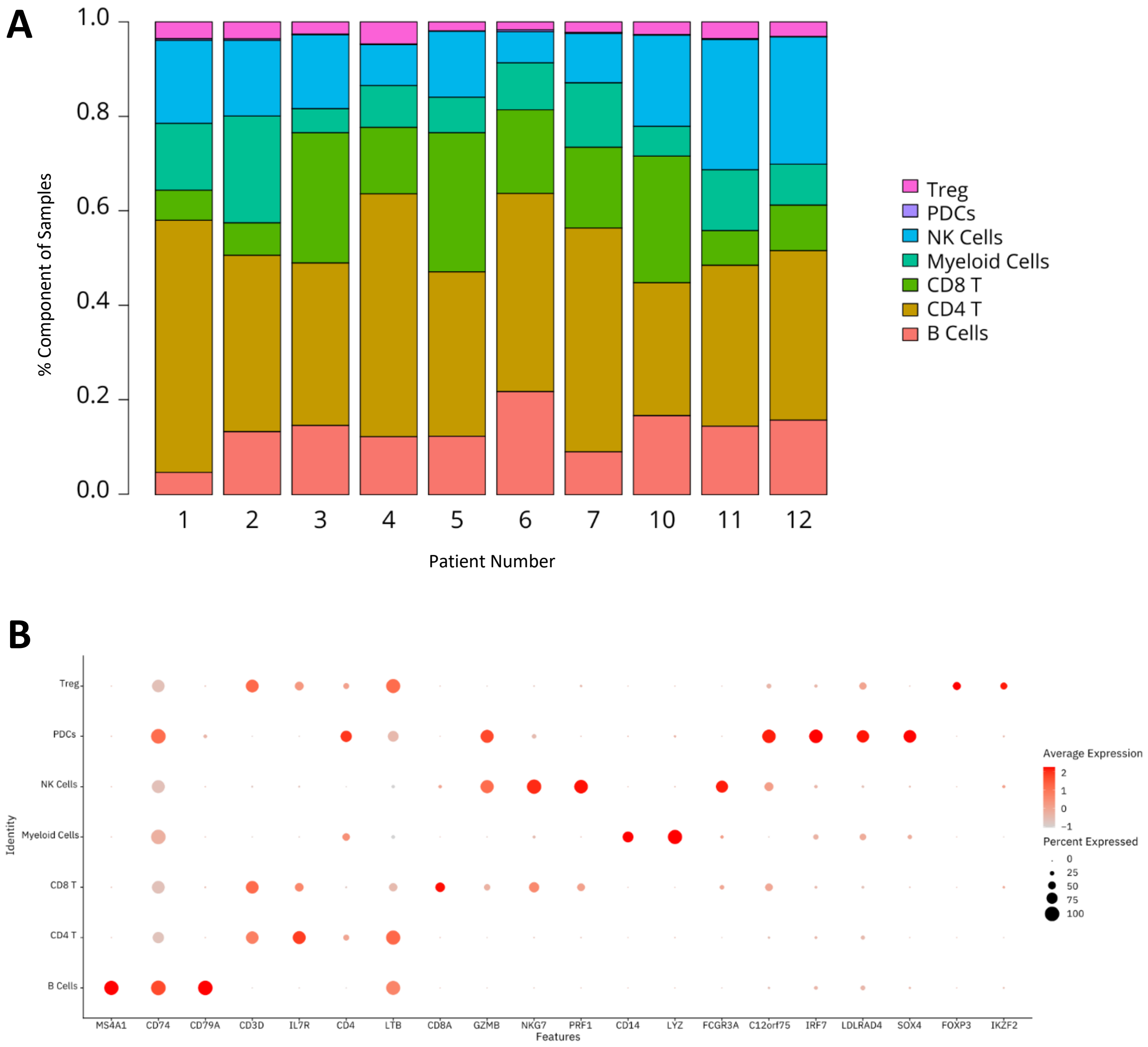

Supplemental Figure 13. (A) Donor-specific cell type distributions within PBMC samples. **(B)** Top marker genes identified per cell type from PBMC samples.

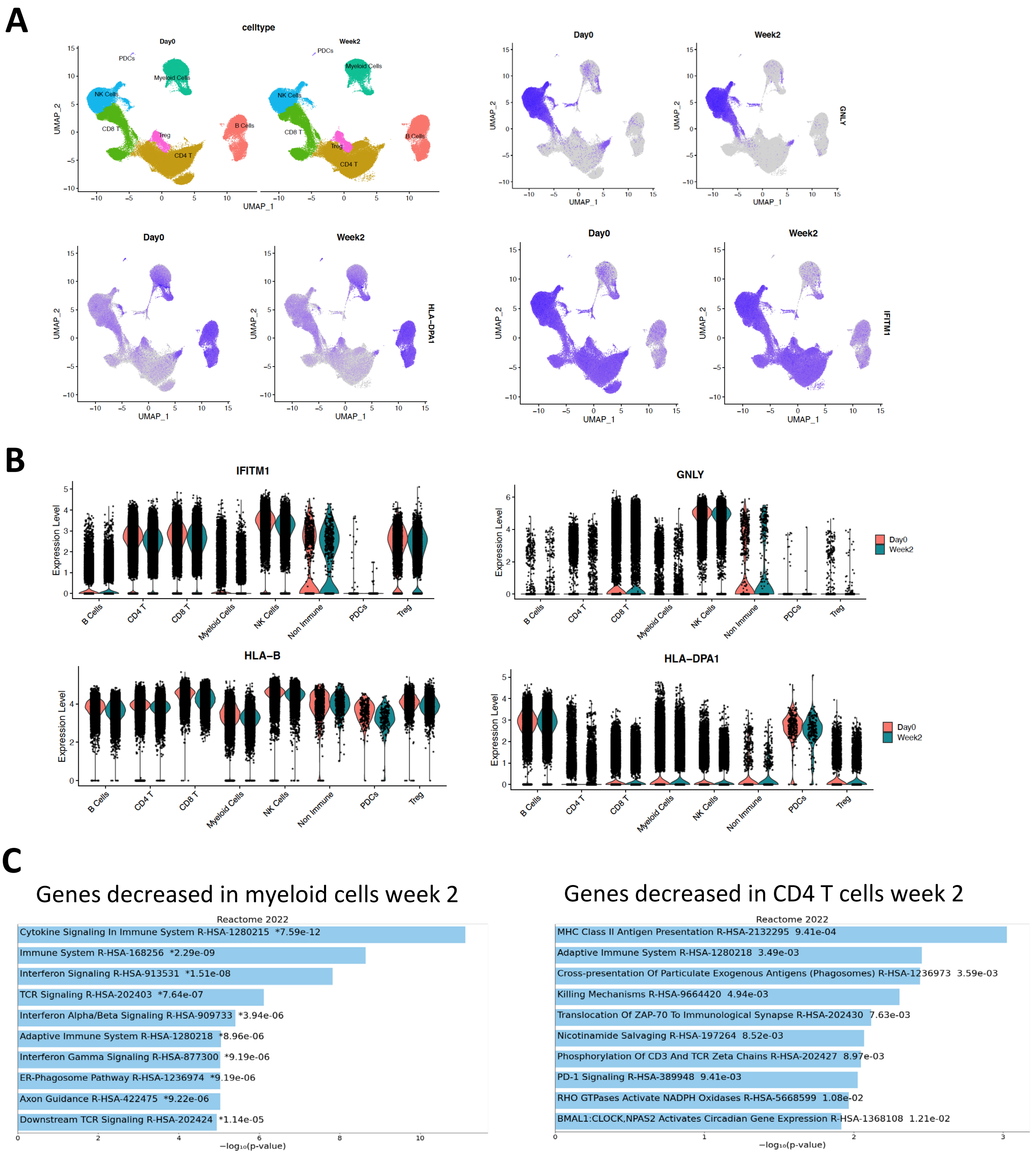

Supplemental Figure 14. (A) Single-cell sequencing of peripheral blood mononuclear cells (PBMCs) at baseline (day 0) and week 2. **(B)** Changes in interferon signature genes (IFITM1), cytotoxic marker (GNLY), MHC class I (HLA-B), and MHC class II (DLA-DPA1) from PBMC samples at baseline (day 0) vs. week 2 of treatment. **(C)** Enriched gene ontology categories amongst DEGs in myeloid cells and CD4 T cells comparing week 2 vs baseline (n=10 at day 0, and n=10 at week 2

Supplemental Note: Trial Eligibility Criteria

Subjects eligible for inclusion in this study must fulfill all the following criteria:

- Subjects must be able to understand and comply with the requirements of the study and communicate with the investigator. Subjects must give written, signed, and dated informed consent before any study related activity is performed. When appropriate, a legal representative will sign the informed consent according to local laws and regulation
- Both men and women must be at least 18 years of age at the time of screening
- Subjects must have clinical and histological features of LP
- LP requiring systemic treatment
- Subjects must have treatment naïve cutaneous LP or treatment refractory disease, as defined by failure of at least one established treatment for LP
    - Failure of prior therapy
        - Topical treatment
        - Systemic immunosuppressant
        - Oral metronidazole
        - Oral sulfasalazine
        - Oral retinoid

Exclusion Criteria

Subjects fulfilling **any** of the following criteria are not eligible for inclusion in this study. To ensure the recruitment of a representative sample of all eligible subjects, the investigator may apply no additional exclusions.

- On excluded therapies, not on a stable dose of a therapy, or incompletely washed out for a therapy.
- Known hypersensitivity or other adverse reaction to Baricitinib (LY3009104)
- Variants of LP deemed by the investigators to be inappropriate for Baricitinib (LY3009104) including but not limited to:
    - Drug-induced LP
    - Predominant non-cutaneous variants of LP, note that individuals can have disease in non-cutaneous areas; however, they must also have cutaneous disease.
        - Lichen Planopilaris
        - Oral Lichen planus
- Pregnant or nursing (lactating) women (pregnancy is defined as the state of a female after conception and until the termination of gestation, confirmed by a positive human chorionic gonadotropin (hCG) laboratory test)
- Women of childbearing potential [Post-menopausal or not of child-bearing potential is defined by 1 year of natural (spontaneous) amenorrhea or surgical bilateral oophorectomy (with or without hysterectomy), total hysterectomy or tubal ligation at least 6 weeks ago. Oophorectomy alone must be confirmed by follow up hormone level assessment to be considered not of child-bearing potential, defined as all women physiologically capable of becoming pregnant, unless they are using basic methods of contraception which includes:
    - Total abstinence (Periodic abstinence and withdrawal are not acceptable methods of contraception)

    - Female sterilization (bilateral oophorectomy with or without hysterectomy), total hysterectomy, or tubal ligation at least 6 weeks before taking study treatment. Oophorectomy alone requires follow up hormone level assessment for fertility.
    - Male sterilization (at least 6 months prior to screening). The vasectomized male partner should be the sole partner for that subject.
    - Barrier methods of contraception: condom or occlusive cap.
    - Use of oral, injected or implanted hormonal methods of contraception or other forms or hormonal contraception that have complete efficacy (failure <1%). (The dose of the contraceptive should be stable for 3 months)
- Active ongoing inflammatory diseases of the skin other than LP that might confound the evaluation of the benefit of Baricitinib (LY3009104)
- Underlying condition (including, but not limited to metabolic, hematologic, renal, hepatic, pulmonary, neurologic, endocrine, cardiac, infectious or gastrointestinal conditions) which, in the opinion of the investigator, significantly immunocompromises the subject and/or places the subject at unacceptable risk for receiving an immunomodulatory therapy
- Moderate-to-severe renal impairment including patients with estimated glomerular filtration rate (eGFR) <60 mL/min/$1.73m^2$
- Active systemic infections during the 2 weeks prior to randomization (common cold viruses excluded) or any infection that reoccurs on a regular basis.
- Current severe progressive or uncontrolled disease which the investigator renders the subject unsuitable for the trial or puts the subject at increased risk
    - Have had any major surgery within 8 weeks prior to screening or will require major surgery during the study that, in the opinion of the investigator would pose an unacceptable risk to the patient.
    - Have experienced any of the following within 12 weeks of screening: VTE (DVT/pulmonary embolism [PE]), myocardial infarction (MI), unstable ischemic heart disease, stroke, or New York Heart Association Stage III/IV heart failure.
    - Have a history of recurrent (≥ 2) VTE (DVT/PE).
    - Have a history of lymphoproliferative disease; have signs or symptoms suggestive of possible lymphoproliferative disease, including lymphadenopathy or splenomegaly; have active primary or recurrent malignant disease; or have been in remission from clinically significant malignancy for <5 years prior to randomization.
    - Have had symptomatic herpes zoster infection within 12 weeks prior to randomization.
    - Have a history of disseminated/complicated herpes zoster (for example, ophthalmic zoster or CNS involvement).
    - ALT or AST >2 x upper limits of normal (ULN); alkaline phosphatase (ALP) ≥2 x ULN; total bilirubin ≥1.5 x ULN; hemoglobin <10 g/dL (100.0 g/L); total white blood cell count <3000 cells/µL (<3.00 x 103/µL or <3.00 billion/L); neutropenia (absolute neutrophil count [ANC] <1500 cells/□L) (<1.50 x 103/□L or <1.50 billion/L); lymphopenia (lymphocyte count <1000 cells/µL) (<1.00 x 103/µL or <1.00 bilion/L); thrombocytopenia (platelets <100,000 cells/µL) (<100 x 103/µL or <100 billion/L)
    - Have a positive test for hepatitis B virus (HBV) defined as:
      a. positive for hepatitis B surface antigen (HBsAg), or
      b. positive for hepatitis B core antibody (HBcAb) and positive for hepatitis B virus deoxyribonucleic acid (HBV DNA)

Note: Patients who are HBcAb-positive and HBV DNA-negative may be enrolled in the study but will require additional HBV DNA monitoring during the study.

- Have hepatitis C virus (HCV) infection (hepatitis C antibody-positive and HCV ribonucleic acid [RNA]-positive).

Note: Patients who have documented anti-HCV treatment for a past HCV infection AND are HCV RNA-negative may be enrolled in the study.

- Have evidence of HIV infection and/or positive HIV antibodies.
- Have had household contact with a person with active TB and did not receive appropriate and documented prophylaxis for TB.
- Have evidence of active TB or latent TB
- Have evidence of active TB, defined in this study as the following:
    - Positive purified protein derivative (PPD) test (≥5 mm induration between approximately 2 and 3 days after application, regardless of vaccination history), medical history, clinical features, and abnormal chest x-ray at screening.
    - QuantiFERON®-TB Gold test or T-SPOT®.TB test (as available and if compliant with local TB guidelines) may be used instead of the PPD test. Patients are excluded from the study if the test is not negative and there is clinical evidence of active TB.

Exception: patients with a history of active TB who have documented evidence of appropriate treatment, have no history of re-exposure since their treatment was completed, have no clinical features of active TB, and have a screening chest x-ray with no evidence of active TB may be enrolled if other entry criteria met. Such patients would not be required to undergo the protocol-specific TB testing for PPD, QuantiFERON®-TB Gold test, or T-SPOT®.TB test but must have a chest x-ray at screening (i.e., chest imaging performed within the past 6 months will not be accepted).

- Have evidence of untreated/inadequately or inappropriately treated latent TB, defined in this study as the following:
    - Positive PPD test, no clinical features consistent with active TB, and a chest x-ray with no evidence of active TB at screening; or
    - If the PPD test is positive and the patient has no medical history or chest x-ray findings consistent with active TB, the patient may have a QuantiFERON®-TB Gold test or T-SPOT®.TB test (as available and if compliant with local TB guidelines). If the test results are not negative, the patient will be considered to have latent TB (for purposes of this study); or
    - QuantiFERON®-TB Gold test or T- SPOT®.TB test (as available and if compliant with local TB guidelines) may be used instead of the PPD test. If the test results are positive, the patient will be considered to have latent TB. If the test is not negative, the test may be repeated once within approximately 2 weeks of the initial value. If the repeat test results are again not negative, the patient will be considered to have latent TB (for purposes of this study).
- Have been exposed to a live vaccine within 12 weeks of randomization or are expected to need/receive a live vaccine during the course of the study (with the exception of herpes zoster vaccination).
- Have donated more than a single unit of blood within 4 weeks prior to screening or intend to donate blood during the course of the study.
- Have a history of intravenous drug abuse, other illicit drug abuse, or chronic alcohol abuse within the 2 years prior to screening or are concurrently using, or expected to use during the study, illicit drugs (including marijuana).
